# Predicted B Cell Epitopes Highlight the Potential for COVID-19 to Drive Self-Reactive Immunity

**DOI:** 10.3389/fbinf.2021.709533

**Published:** 2021-08-19

**Authors:** Rhiane Moody, Kirsty L. Wilson, Jennifer C. Boer, Jessica K. Holien, Katie L. Flanagan, Anthony Jaworowski, Magdalena Plebanski

**Affiliations:** ^1^ School of Health and Biomedical Science, STEM College, RMIT University, Bundoora, VIC, Australia; ^2^ School of Science, STEM College, RMIT University, Bundoora, VIC, Australia; ^3^ Tasmanian Vaccine Trial Centre, Clifford Craig Foundation, Launceston General Hospital, Launceston, TAS, Australia; ^4^ School of Medicine, University of Tasmania, Launceston, TAS, Australia; ^5^ Department of Immunology and Pathology, Monash University, Melbourne, VIC, Australia

**Keywords:** peptides, epitope mapping, COVID-19, self-reactivity, molecular-mimicry, autoimmunity

## Abstract

COVID-19, caused by the Severe Acute Respiratory Syndrome Coronavirus 2 (SARS-CoV-2), whilst commonly characterised as a respiratory disease, is reported to have extrapulmonary manifestations in multiple organs. Extrapulmonary involvement in COVID-19 includes autoimmune-like diseases such as Guillain-Barré syndrome and Kawasaki disease, as well as the presence of various autoantibodies including those associated with autoimmune diseases such a systemic lupus erythematosus (e.g. ANA, anti-La). Multiple strains of SARS-CoV-2 have emerged globally, some of which are found to be associated with increased transmissibility and severe disease. We performed an unbiased comprehensive mapping of the potential for cross-reactivity with self-antigens across multiple SARS-CoV-2 proteins and compared identified immunogenic regions across multiples strains. Using the Immune Epitope Database (IEDB) B cell epitope prediction tool, regions predicted as antibody epitopes with high prediction scores were selected. Epitope sequences were then blasted to eight other global strains to identify mutations within these regions. Of the 15 sequences compared, eight had a mutation in at least one other global strain. Predicted epitopes were then compared to human proteins using the NCBI blast tool. In contrast to studies focusing on short sequences of peptide identity, we have taken an immunological approach to selection criteria for further analysis and have identified 136 alignments of 6–23 amino acids (aa) in 129 human proteins that are immunologically likely to be cross-reactive with SARS-CoV-2. Additionally, to identify regions with significant potential to interfere with host cell function-or promote immunopathology, we identified epitope regions more likely to be accessible to pathogenic autoantibodies in the host, selected using a novel combination of sequence similarity, and modelling protein and alignment localization with a focus on extracellular regions. Our analysis identified 11 new predicted B-cell epitopes in host proteins, potentially capable of explaining key aspects of COVID-19 extrapulmonary pathology, and which were missed in other *in silico* studies which used direct identity rather than immunologically related functional criteria.

## Introduction

In March 2020, the World Health Organization (WHO) declared the disease known as COVID-19, caused by the Severe Acute Respiratory Syndrome Coronavirus (SARS-CoV-2), as a global pandemic. As of the 2nd of August 2021, there have been over 198.2 million confirmed cases and over 4.3 million deaths reported (WHO, 2020a). COVID-19 patients typically present with symptoms such as fever, dry cough, tiredness, as well as others such as myalgia, sore throat, loss of taste or smell, red eyes, diarrhoea and skin rash (Huang et al., 2020; Meng et al., 2020; WHO, 2020b). The SARS-CoV-2 genome encodes a series of structural and non-structural proteins (Chan et al., 2020; Wu et al., 2020). There are four structural proteins consisting of the surface glycoprotein (spike, SP), nucleocapsid phosphoprotein (nucleoprotein, NP), membrane (M) and envelope (E). There are additionally eight open reading frames (Orf) encoding the non-structural proteins: Orf1ab, Orf3a, Orf3b, Orf6, Orf7a, Orf7b, Orf8 and Orf10. Multiple studies have used serological assays to measure antibody responses to the SARS-CoV-2 virus (Long et al., 2020; Zhao et al., 2020; Post et al., 2021). Antibodies are usually evaluated for reactivity against the spike and the nucleoprotein with the aim of understanding seroconversion, as well as correlating disease severity with neutralizing antibody titres (systematically reviewed in Post et al. (2021)). Additional reports have identified increases in antibodies to other SARS-CoV-2 proteins, including Orf proteins, after infection (Hachim et al., 2020). Since the beginning of the pandemic, variants of SARS-CoV-2 have been emerging and circulating worldwide (van Dorp et al., 2020). Some mutations, such as K417N and E484K in the spike protein, are immune evasion mutations (Altmann et al., 2021). Since the second half of 2020 variants of SARS-CoV-2, containing mutations such as these, have arisen with increased transmissibility, associated with more severe disease and a reduction in neutralizing antibodies. These variants have been called variants of concern (VOC) or variants of interest (VOI). As of July 2021, there are four VOCs and four VOIs classified by the Centers for Disease Control and Prevention (CDC) (SARS-, 2021). The VOCs consist of the strains originating in the United Kingdom (Alpha strain), Brazil (detected in Japan, Gamma strain), South Africa (Beta strain), and India (Delta strain). Whereas VOIs consist of strains originating in Canada (Eta strain), United States (Iota strain), India (Kappa strain) and Peru (Lambda strain).

Viral infections generally can play a role in promoting or exacerbating autoimmune diseases (reviewed in Smatti et al. (2019)). Proposed mechanisms for this association include molecular mimicry and bystander activation (Fujinami et al., 2006; Smatti et al., 2019). Molecular mimicry refers to the phenomenon of immune cross-reactivity between a foreign pathogen and self-protein, where an immune cell recognizes both due to their sequence similarity (Fujinami et al., 2006; Smatti et al., 2019). Bystander activation refers to the activation of self-reacting immune cells driven by the liberation of self-antigens (the targets which stimulate immune responses) from virus-lysed cells which otherwise would not be typically exposed to the immune system (Fujinami et al., 2006; Smatti et al., 2019). Given that a range of extrapulmonary pathologies have been reported in COVID-19 (Cheng et al., 2020; Cheung et al., 2020; Han et al., 2020; Hundt et al., 2020; Nalleballe et al., 2020; Oxley et al., 2020; Poyiadji et al., 2020; Xie et al., 2020; Zheng et al., 2020), molecular mimicry has been hypothesized to be playing a role (Angileri et al., 2020a; Cappello et al., 2020; Marino Gammazza et al., 2020). Additionally, conditions similar to the autoimmune diseases Guillain-Barré Syndrome (Ameer et al., 2020; Korem et al., 2020) and Kawasaki disease (Bordet et al., 2020; Jones et al., 2020) have been reported in COVID-19 patients. Within this Kawasaki-like disease, termed Multisystem Inflammatory Syndrome in children (MIS-C), autoantibodies targeting a range of antigens including, but not limited to, anti-La, anti-Jo-1, anti-MUC15, anti-P2RX4, anti-MAP2K2 and anti-CSNK1A1 have been reported (Consiglio et al., 2020; Gruber et al., 2020). Additionally, autoantibodies targeting other antigens including type I interferons (IFNs) (Bastard et al., 2020) have been identified in COVID-19 positive patients (Zhou et al., 2020a; Bastard et al., 2020; Vlachoyiannopoulos et al., 2020) alongside key autoimmune associated antigens which are often associated with tissue damage, for example, anti-nuclear antigen (ANA) (Zhou et al., 2020a; Vlachoyiannopoulos et al., 2020). It is therefore highly likely autoantibodies that recognize a range of other self-proteins are induced by infection with SARS-CoV-2.

Computational biology methods such as immune epitope mapping and Basic Local Alignment Search Tool (BLAST) allow for relatively quick predictive screening analysis. They can help narrow findings and hypotheses, leading to more targeted laboratory-based validation work. In the context of COVID-19, understanding immune epitopes, the sequences that are recognized by antibodies, have been used to predict potential peptide-based vaccine candidates or to increase sensitivity in serological assays (Ahmed et al., 2020; Amrun et al., 2020; Poh et al., 2020). In the initial stages of the COVID-19 outbreak, B-cell epitope mapping was performed in SARS-CoV-2 to check for potential cross-reactivity of immune responses with other coronaviruses (Ahmed et al., 2020; Grifoni et al., 2020). Recently, there have been a small number of studies reporting sequence similarities between SARS-CoV-2 proteins and human proteins, in support of the molecular mimicry hypothesis (Angileri et al., 2020a; Angileri et al., 2020b; Ehrenfeld et al., 2020; Kanduc, 2020; Kanduc and Shoenfeld, 2020; Lucchese and Flöel, 2020; Lyons-Weiler, 2020; Marino Gammazza et al., 2020). With the exception of one study, which used longer epitopes (Lyons-Weiler, 2020), most of these studies sought to find short regions (5-6aa) of exact identity between the SARS-CoV-2 proteins and human proteins (Angileri et al., 2020a; Angileri et al., 2020b; Kanduc, 2020; Kanduc and Shoenfeld, 2020; Lucchese and Flöel, 2020; Marino Gammazza et al., 2020). Whilst these studies suggested a number of regions, most of the identical sequences identified would be unlikely to sustain a pathogenic autoreactive response (Angileri et al., 2020a; Kanduc, 2020; Kanduc and Shoenfeld, 2020; Lucchese and Flöel, 2020; Marino Gammazza et al., 2020). Specifically, while assessing its potential accessibility for antibody binding, these studies did not discriminate the localization of the epitope within the protein or within the cell (Angileri et al., 2020a; Kanduc, 2020; Kanduc and Shoenfeld, 2020; Lucchese and Flöel, 2020; Lyons-Weiler, 2020; Marino Gammazza et al., 2020). Moreover, except in the case of Kanduc (2020), who identified identical hexamers between SARS-CoV-2 and human proteins associated with various system disorders, these studies only report small numbers of human proteins with similar (Lyons-Weiler, 2020) or short identical sequences (Angileri et al., 2020a; Kanduc and Shoenfeld, 2020; Lucchese and Flöel, 2020; Marino Gammazza et al., 2020) to SARS-CoV-2 proteins (e.g. association with the immune system, molecular chaperones, brainstem proteins). Therefore, the limited nature of these studies does not show the full extent of similarities between SARS-CoV-2 and human proteins.

In the present study we have pioneered a different immunoinformatics approach to overcome some of these limitations and further explore the potential for autoimmune cross-reactivity driven by SARS-CoV-2 molecular mimicry. B-cell epitopes within the structural proteins and several Orf proteins (shown to generate antibody responses in COVID-19 positive patients (Hachim et al., 2020)), were first predicted using the IEDB B Cell epitope tool. Epitopes predicted in the structural proteins were compared to the current VOCs (Alpha, Beta, Gamma and Delta) and VOIs (Eta, Iota, Kappa and Lambda). Eight of the fifteen epitopes contained at least one mutation in one variant. The identified 9-53aa long sequences, in both structural and Orf proteins, were then compared to sequences in human proteins using the NCBI protein BLAST tool (Altschul et al., 1990). Assessment of potential for cross-reactivity between SARS-CoV-2 and self-proteins with capacity to perpetuate autoimmune pathology was based on a combination of immunologically relevant sequence similarity (not just identity) (Angileri et al., 2020a; Kanduc, 2020; Lucchese and Flöel, 2020; Marino Gammazza et al., 2020) and the localization of the protein itself, with a focus on extracellular targets. 11 human proteins, containing amino acid sequences similar to nine predicted SARS-CoV-2 B cell epitopes, were identified based on these selection criteria. These findings indicate that antibodies induced by SARS-CoV-2 could directly interfere with cell function, including that of immune cells, and could help explain some of the additional pathologies identified in COVID-19 patients (Cheng et al., 2020; Cheung et al., 2020; Han et al., 2020; Hundt et al., 2020; Nalleballe et al., 2020; Oxley et al., 2020; Poyiadji et al., 2020; Xie et al., 2020; Zheng et al., 2020). Finally, comparing the sequences of both predicted spike epitopes and full length spike protein to various human proteins implicated in immune thrombocytopenia purpura (ITP) and thrombocytopenia syndrome (TTS), our results indicate that molecular mimicry is unlikely to be the cause of TTS, or vaccine induced prothrombotic immune thrombocytopenia (VIPIT) following vaccination with the COVID-19 adenovirus vector vaccines. To our knowledge, this is the first study to compare immune epitopes across the circulating VOCs and VOIs; highlight multiple similarities between the selected Orf proteins and human proteins; identify proteins with reported associations to autoimmunity as sharing sequences with SARS-CoV-2 epitopes; and to highlight novel extracellular human proteins which may have antibody cross-reactivity with SARS-CoV-2 immunogenic regions.

## Methods

### Research Pipeline to Explore Potential Immune Cross-Reactivity

**FIGURE 1:**
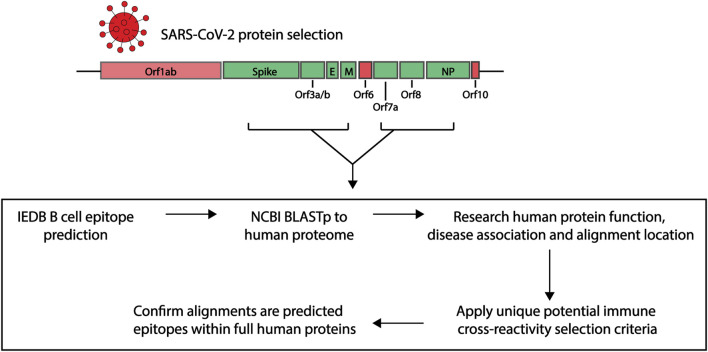
Research pipeline of the project to explore the potential of immune cross-reactivity. Protein sequences for select SARS-CoV-2 proteins were obtained for epitope predictions (highlighted green. Spike: Surface glycoprotein. E, envelope; M, membrane; NP, nucleoprotein). Using the Immune Epitope Database (IEDB) epitope prediction tool, B cell epitopes were predicted and those selected were screened against human proteins using the NCBI Blastp tool. Human proteins with sequence similarities to the SARS-CoV-2 epitopes were investigated for their function, disease association and alignment localization. Potential immune cross-reactivity between SARS-CoV-2 and human alignments was then explored by applying specific selection criteria. Alignments of interest from the human proteins were explored as potential epitopes within the human protein sequences.

In order to explore the similarities between potential immunogenic SARS-CoV-2 regions and human proteins, we applied a research pipeline as outlined in Figure 1. B cell epitopes were first predicted within a selection of SARS-CoV-2 proteins. Those selected were then compared to the human proteome. To explore potential immune cross-reactivity, we applied novel criteria that not only considered short identical sequences but also amino acid variations with conserved structural and charge changes that may not impact antibody binding. We also considered protein localization, with a focus on extracellular proteins. The alignments of interest found within human proteins were cross-checked as potential epitopes within their own protein sequence. Details of each step described below.

### Sequence Identification and Epitope Mapping

Based on previous mapping and assay-based studies (Hachim et al., 2020; Grifoni et al., 2020), epitope prediction was carried out on proteins previously identified from the SARS-CoV-2 isolate Wuhan-hu-1 (Figure 1; Supplementary Table S1) (GenBank: MN908947.3) (Wu et al., 2020). As Orf3b is produced by a premature stop codon (Gordon et al., 2020; Lam et al., 2020) and the sequence could not be identified on GenBank, the sequence provided in Lam et al. (2020) was used for epitope mapping.

Linear B cell epitope predictions were performed using the B cell Epitope prediction tool found within Immune Epitope Database and Analysis Resource (IEDB, http://tools.iedb.org/bcell/ (Dhanda et al., 2019)). Using the Bepipred Linear Epitope Prediction algorithm (Haste Andersen et al., 2006; Larsen et al., 2006; Ponomarenko and Bourne, 2007), predicted epitopes for structural proteins (Spike, Envelope, Membrane and Nucleoprotein), were selected based on prediction score (>1) and having length greater than 6aa. Epitopes for the Orf proteins were predicted using the Bepipred Linear Epitope Prediction 2.0 algorithm (Jespersen et al., 2017) and were selected for having the highest peak points (prediction score >0.5) of length greater than 6aa. Each algorithm was selected based on the IEDB recommended at the time epitope mapping was performed, and prediction score cut offs to select for those more likely to be real epitopes.

Where possible, protein structures were downloaded from the protein data bank rcsb.org (Berman et al., 2000). For SARS-CoV-2 proteins, where regions were uncrystallised, homology models were created using the MPI Bioinformatics Toolkit (https://toolkit.tuebingen.mpg.de/). Specifically, templates were found using HHpred and models were created using Modeller (Zimmermann et al., 2018). Any unstructured regions were deleted and figures were created in The PyMOL Molecular Graphics System, Version 2.0 Schrödinger, LLC. All available human protein models were downloaded from the SWISS-MODEL Repository (Bienert et al., 2017).

### Sequence Alignment to Other SARS-CoV-2 Variants

For SARS-CoV-2 variants protein alignment, we used eight different variants and the original Wuhan sequence (Supplementary Table S2). The FASTA sequences were retrieved from the GISAID database (https://www.gisaid.org/). In the EpiCov search section of the GISAID database there is an available tab that allows for selection of the major circulating variants. Of these we selected the current VOCs (Alpha, Beta, Gamma and Delta) and VOIs (Eta, Iota, Kappa and Lambda). The virus names listed in Supplementary Table S2 are GISAID nomenclature and the specific viruses were selected based on various conditions: for all variants we selected the conditions in which the sequence was complete, we excluded sequences with low coverage (>5% unidentified amino acids) and selected the specific clad of the variant being analyzed. For all variants, each one was chosen within a specific date based on their historical appearance. From these we selected the ones that underwent Illumina sequencing and for which the assembly method was clearly reported. In order to obtain the specific amino acid sequences of the various genomic regions based on the B cell epitope prediction, we retrieved the FASTA sequence of the entire viral genome from the selected variants and blasted these using BlastN by selecting standard databases and optimizing for highly similar sequences (megablast). Since not all submissions to GISAID have also been submitted to NCBI some did not exhibit 100% sequence identity. The percentage of sequence identity for each variant is listed in the table below (column NCBI blast identity in Supplementary Table S2). For the ones that exhibited lower than 100% sequence identity, with blastN we visualized the areas in which the differences were laying. Those specific sequences were then blasted to view where in the viral genome they were appearing.

Using JalView (V2.11.1.4) (Waterhouse et al., 2009), we aligned the amino acid sequences for the surface glycoprotein, membrane protein and nucleoprotein areas of all eight variants plus the original Wuhan sequence, and set the latter as reference genome. We then performed alignment using the Multiple Sequence Alignment using Fast Fourier Transform (MAFFT) alignment program with default settings (V7.110) (Katoh and Standley, 2013). MAFFT is a high speed multiple sequence alignment program which utilizes the Fast Fourier Transform to optimize protein alignments based on the amino acid physical properties.

### Sequence BLAST to Compare Epitopes to Human Proteins and Identification of Associated Diseases, Expression Location and Function

Using the NCBI protein-protein BLAST (blastp, https://blast.ncbi.nlm.nih.gov/ (Altschul et al., 1990)), predicted epitope sequences were blasted for the top 100 results, to the non-redundant protein sequences (nr) database, specifically looking at *Homo sapiens* (Taxid ID: 9606). All other algorithm parameters remained as default settings, including: automatically adjust parameters for short input sequences, and expect threshold = 0.05. Resulting lists were narrowed by excluding isoform repeats, identical proteins with different nomenclature, uncharacterized/hypothetical proteins and variable regions of B and T cell receptors. Associations of the self-proteins to diseases, their expression locations and functions were identified using UniProtKB, GeneCards and PubMed Resources. Any proteins identified through computational analysis or found on an unreviewed UniProtKB database page were further excluded.

For any direct epitope to full protein comparison, “align two or more sequences” in the NCBI blastp tool was used.

### Prediction of Potential Immune Cross-Reactivity Selection Criteria

Using the previously narrowed BLAST lists, potential B cell epitope cross-reactivity was identified based on the following criteria:

1) Covering at least six consecutive amino acids.
2) Where there were amino acid differences (including those interrupting a 6aa consecutive sequence), the similarity between the amino acid structure and charge was compared. Any variances that may impact antibody function or binding, such as structural and charge changes, were excluded.
3) Expression location or alignment region found in the extracellular domain.

### Confirmation of B Cell Epitope Within the Self-Proteins

Complete protein sequences were obtained from the UniProtKB database and mapped for potential B cell epitopes using the IEDB B cell epitope prediction tool. To correspond to the prediction algorithm used for the SARS-CoV-2 proteins, the Bepipred Linear Epitope Prediction algorithm (Haste Andersen et al., 2006; Larsen et al., 2006; Ponomarenko and Bourne, 2007) was used for human proteins where the shared alignments were to structural SARS-CoV-2 proteins. Whereas proteins which shared alignment with non-structural Orf proteins were mapped using the Bepipred Linear Epitope Prediction 2.0 algorithm (Jespersen et al., 2017). To confirm if any epitopes had been validated, the UniProtKB ID was used to search the antigens and epitopes in the IEDB database (Vita et al., 2019).

## Results

### Identification and Selection of B Cell Epitopes in SARS-CoV-2 Proteins

**FIGURE 2:**
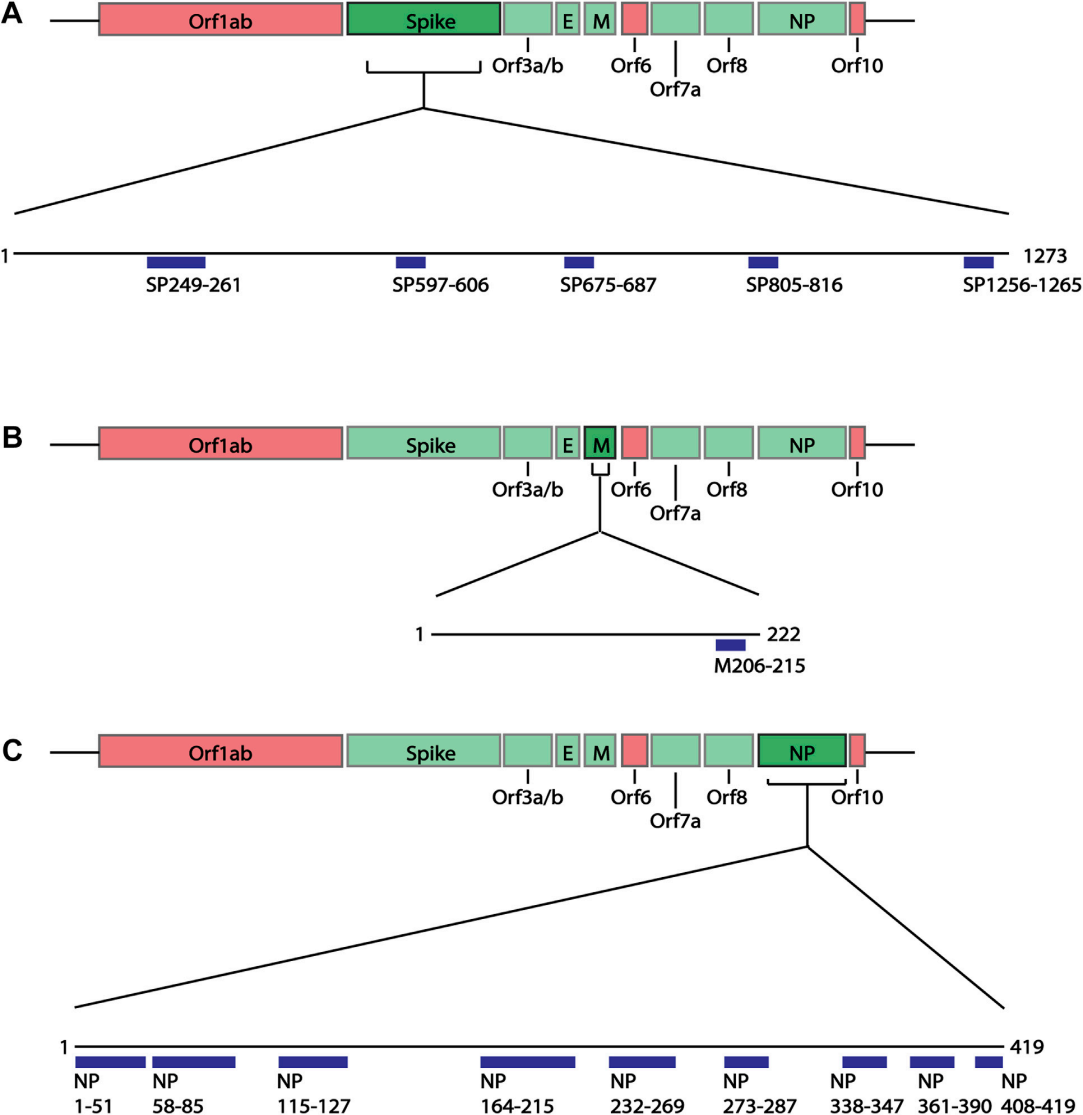
Linear schematic of selected B cell epitopes in SARS-CoV-2 Structural proteins. B cell epitopes within the SARS-CoV-2 spike (SP), membrane (M) and nucleoprotein (NP) were mapped and selected based as having ≥6aa length and a predicted epitope score ≥1. **(A)** Five epitopes in the spike protein, beginning at positions aa249, 597, 675, 805 and 1256 were selected for downstream analysis. **(B)** One epitope in the membrane protein at position aa205 was identified. **(C)** Nine epitopes in the nucleoprotein, aa1, 58, 115, 164, 232, 273, 338, 361, 408 were identified. All associated sequences can be found in Supplementary Table S3.

**FIGURE 3:**
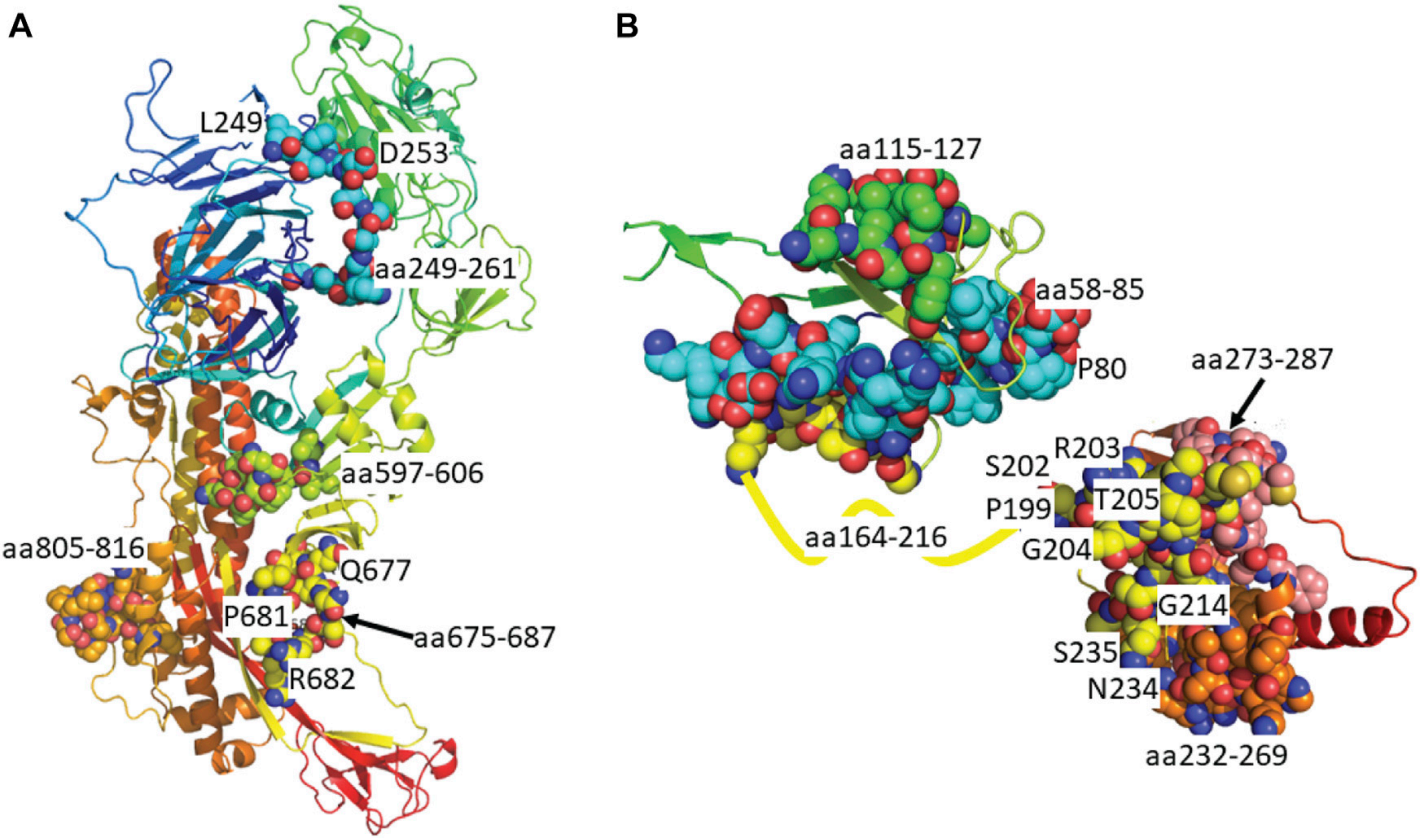
Predicted epitopes mapped to protein structure of Surface Glycoprotein and Nucleoprotein. **(A)** X-ray crystal structure of the Surface Glycoprotein (Cai et al., 2020) coloured *via* spectrum (N to C terminal) with the structured B cell epitopes highlighted *via* spheres; aa294-261 (cyan), aa597-606 (green), aa675-687 (yellow) and aa805-816 (orange). Also labelled are the mutation sites; L249, D253, Q677, P681 and R682. **(B)** Structure of the nucleoprotein coloured *via* spectrum (N to C terminal). The N-terminal regions are from the X-ray crystal structure (PDB code: 6vyo) followed by an unstructured linear (yellow line) to a C-terminal homology model. The structural B cell epitopes are highlighted *via* spheres; aa58-85 (cyan), aa115-127 (green), aa164-216 (yellow), aa232-269 (orange) and aa273-287 (salmon). Also labelled is the location of known mutation sites; P80, P199, R201, S202, R203, T205, G214, N234 and S235.

As previous studies have looked at the structural proteins (Grifoni et al., 2020), B cell epitope mapping was initially performed on the four structural proteins of SARS-CoV-2 Wuhan-hu-1 variant: spike (SP), membrane (M), envelope (E) and nucleoprotein (NP), using the IEDB B cell Epitope Prediction Tool. In B cell epitopes, 4-6aa often form the minimal binding region (Pieczenik, 2003), and most B cell epitopes have a length between 4 and 25aa (Gupta et al., 2013; Potocnakova et al., 2016) (but can be as long as 50aa (Gupta et al., 2013)). As some epitopes are composed of discontinuous multiple linear segments of 1-6aa (Rubinstein et al., 2008), we selected the predicted epitopes of 6aa or more. Additionally, we applied a cut off of at least score 1, selecting those specifically with the higher prediction score and therefore more likely to be real epitopes. Based on these criteria, we identified 15 epitopes of interest ranging between 10 and 53aa (Figure 2; Supplementary Table S3): five spike epitopes of which two are C-terminally unstructured (Figure 3A), one unstructured C-terminal membrane epitope, and nine nucleoprotein epitopes of which two are partially structured, and three are C-terminal unstructured motifs (Figure 3B). During the course of the present study, four of the five spike epitopes (249-261, 597-606, 805-816, 1256-1265), and three of the nine nucleoprotein epitopes (164-216, 232-269, 361-390) we predicted, were further shown by others to be regions which overlap with new epitopes identified in laboratory studies (Amrun et al., 2020; Poh et al., 2020; Shrock et al., 2020; Wang et al., 2020; Yi et al., 2020; Yoshida et al., 2021), providing support to the selected epitope mapping approach.

**FIGURE 4:**
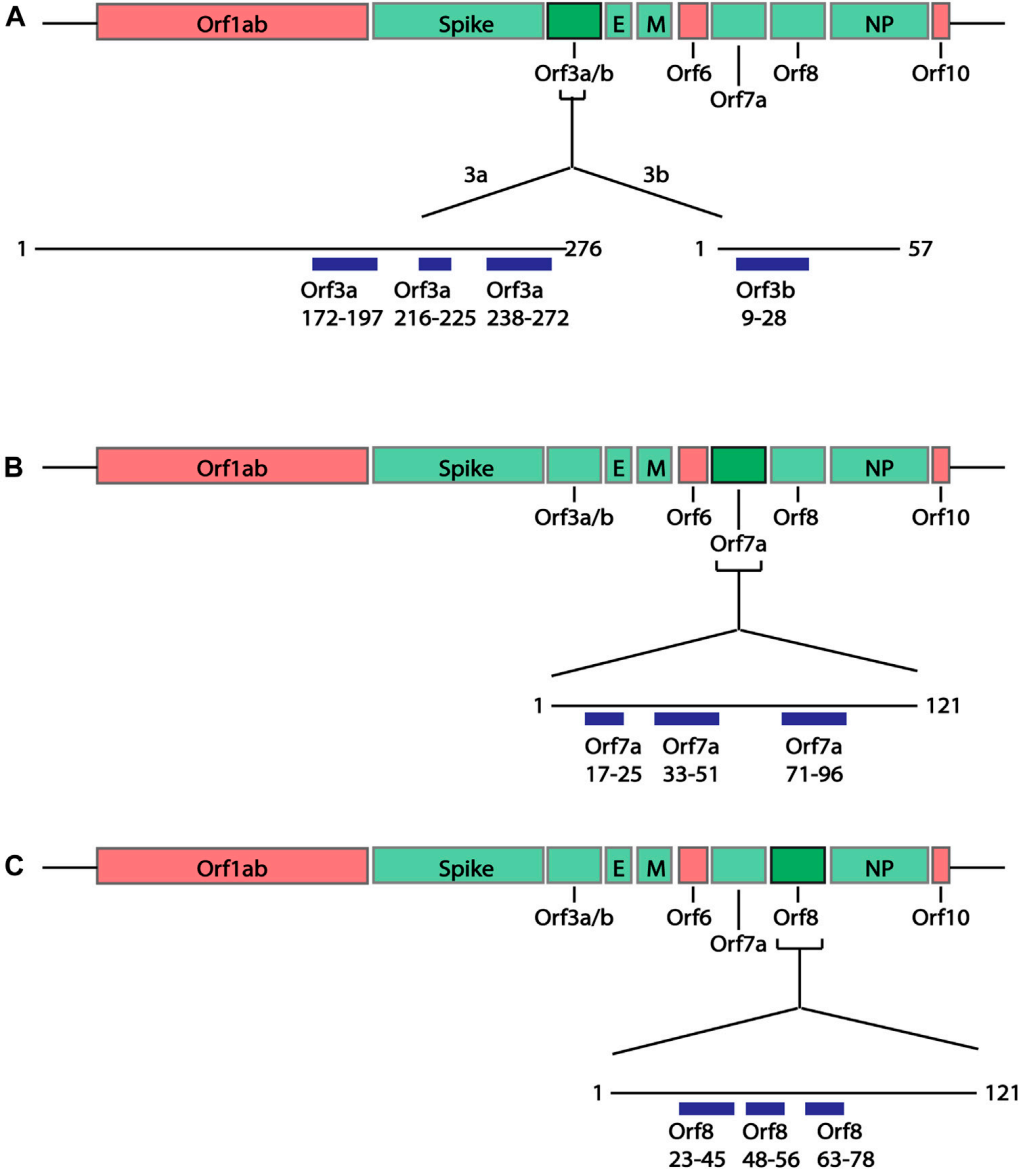
Linear schematic of selected B cell epitopes in SARS-CoV-2 Orf proteins. B cell epitopes within the SARS-CoV-2 Orf3a, Orf3b, Orf7a and Orf8 and selected based as having ≥6aa length and a predicted epitope score ≥0.55. **(A)** Three epitopes in Orf3a (aa172-197, 216-225 and 238-272) and one epitope in Orf3b (aa9-28) were identified. **(B)** Three epitopes in Orf7a (aa17-25, 33-51 and 71-96) were identified. **(C)** Three epitopes in Orf8 (aa23-45, 48-56 and 63-78). All associated sequences can be found in Supplementary Table S3.

**FIGURE 5:**
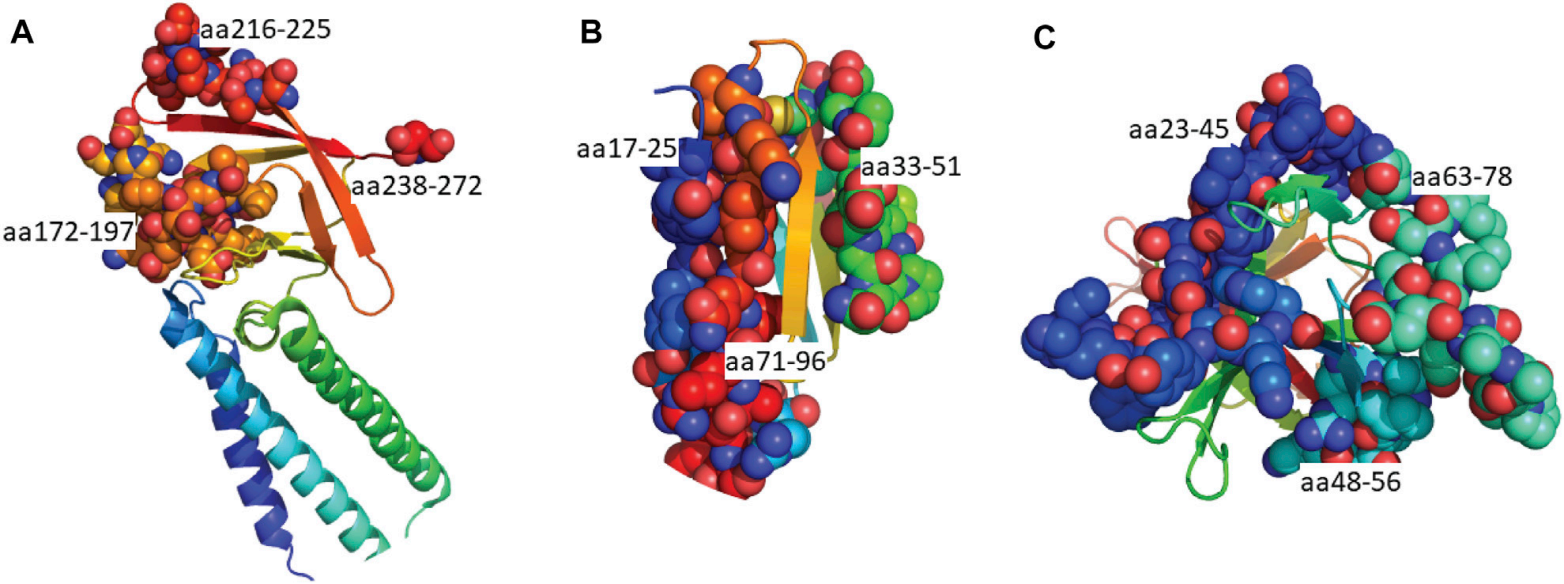
Predicted epitopes mapped to the structures of SARS-CoV-2 Orf proteins. **(A)** Cryo-EM structure of the Orb3a (Kern et al., 2021) coloured via spectrum (N to C terminal) with the structured B cell epitopes highlighted *via* spheres; aa172-197 (orange), aa216-225 (light red), aa238-272 (red). **(B)** X-ray crystal structure of Orb7a (Zhou et al., 2021) with the structured B cell epitopes highlighted *via* spheres; aa17-25 (blue), 33-51 (green) and aa71-96 (red). **(C)** X-ray crystal structure of Orb7a (Flower et al., 2021) with the structured B cell epitopes highlighted *via* spheres; aa 23-45 (blue), 1148-56 (cyan) and aa63-78 (light green).

In addition to the structural proteins, epitopes were also predicted for Orf3a, Orf3b, Orf7a and Orf8 SARS-CoV-2 proteins since antibodies against these proteins have been identified in COVID-19 patients (Hachim et al., 2020). Similar to the structural proteins, epitope sequences were selected to have a minimal length of 6aa. As the Bepipred v2.0 algorithm (Jespersen et al., 2017) was used to predict these epitopes, the prediction score criteria were modified to have a score greater than 0.55, representing the higher predicted immunogenic regions in these proteins shown to have antibody responses. Based on these criteria a total of 10 epitopes were identified within the Orf proteins (Figure 4; Supplementary Table S3); two partially structured, one C-terminal partially structure epitope for Orf3a (Figure 5A), three structured epitopes for Orf7a and Orf8 (Figures 5B,C, respectively) and a single unstructured N-terminal epitope within Orf3b. Of these, sequences Orf3a 172-197, Orf3a 216-225, Orf8 23-45 and Orf8 48-56 have been validated to be within a region to which COVID-19 patients have antibody responses (Shrock et al., 2020; Wang et al., 2020).

### Comparison of Predicted Immunogenic Regions Among SARS-CoV-2 Variants of Concern and Variants of Interest

**FIGURE 6:**
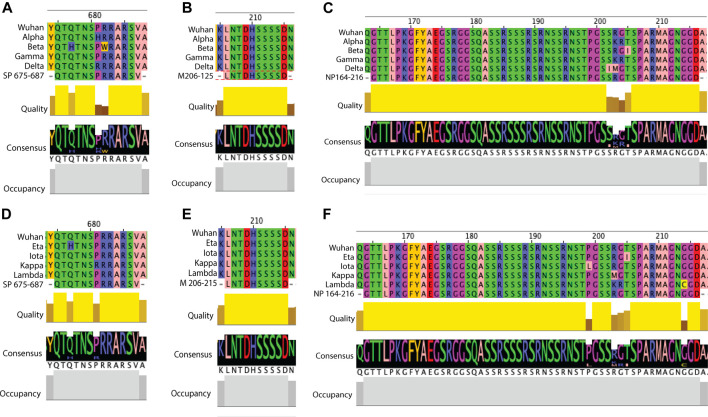
Examples of sequence comparison between predicted B cell epitopes and key SARS-CoV-2 variants. Structural protein sequences for a strain from Wuhan (WIV04/2019), VOCs (Alpha, Beta, Gamma and Delta) and VOIs (Eta, Iota, Kappa and Lambda) were obtained from the GISAID database and blast aligned with predicted B cell epitopes. Quality refers to the alignment quality based on blosum2 algorithm scores, Consensus indicates the abundance of the amino acids present in a particular position and Occupancy is the number of aligned positions. **(A)** SP VOC sequence alignment at position aa675-687. **(B)** VOC sequence alignment of membrane aa206-215. **(C)** NP VOC sequence alignment at position aa164-216. **(D)** SP VOI sequence alignment at position aa675-687. **(E)** VOI sequence alignment of membrane aa206-215. **(F)** NP VOI sequence alignment at position aa164-216.

With the multiple variants of concern and of interest circulating worldwide which can escape neutralization and cause more severe disease (Garcia-Beltran et al., 2021), we were interested in whether these predicted B cell epitopes overlapped with known escape mutations or contain any mutations which may impact antibody responses. As the antibody responses to structural proteins, specifically the spike and nucleoprotein, have been extensively studied and are used for serological assays (Post et al., 2021), potential mutations in these proteins were of interest. Using the GISAID database to obtain sequences for the VOCs: Alpha, Beta, Gamma and Delta variants, the structural proteins (spike, membrane and nucleoprotein) were blast aligned with predicted epitopes to identify any mutations in these regions (Supplementary Figures S1, S2; Supplementary Table S4). Of the five predicted spike epitopes only SP675-687 contained any mutation (Figures 3A, 6A). In this 13aa sequence the Beta variant contains two mutations, Q677H and R682W. At position 681, both the Delta and Alpha variants contained mutations P681R and P681H respectively. In each case, these mutations consist of a structural and/or charge mutation which may impact antibody binding. For example, the P681 mutations would be expected to increase flexibility in this region. No mutation was identified within the one membrane epitope predicted (Figure 6B). Among the nine nucleoprotein epitopes, NP115-127, 273-287, 338-347 and 408-419 did not contain any mutations. NP1-51 contained a mutation in the Alpha variant at D3L. Epitopes NP58-85, 232-269 and 361-390 each only have one mutation: P80R (Gamma), S235F (Alpha) and D377Y (Delta). Multiple mutations were identified in NP164-216 (Figures 3B, 6C). The Delta variant contained two mutations S202I and R203M. Position 203 was additionally mutated in the Alpha and Gamma variants, R203K. These variants were also mutated at position 204, G204R. There was additionally a mutation in the Beta variant, T205I. Except for mutations R203K and T205I in NP164-216, all mutations identified result in a structural and/or charge change that may impact antibody binding, resulting in the more severe disease seen in these variants.

In addition to looking at the VOCs, we further looked into whether any mutations occurred within the predicted epitopes in the VOIs: Eta, Iota, Kappa and Lambda (Supplementary Figures S3, S4; Supplementary Table S4). Within the five predicted spike epitopes, SP249-261 and SP675-687 contained mutations (Figure 6D). In SP249-261, the Lambda variant was missing aa249-252 and contained the mutation D253N. At this same residue the Iota variant had the mutation D253G. In each of these cases a structural and or charge change occur. Both Eta and Kappa variants contained a mutation in SP675-687, Q677H and P681R, respectively. Each of these are shared mutations in VOCs Beta and Delta, respectively. As seen in the VOCs, no mutation was identified in the single predicted membrane epitope (Figure 6E). Within the nine predicted nucleoprotein epitopes, NP1-51, NP164-216, NP232-269 and NP351-390 contain mutations. The Eta variant contained four mutations in NP1-51: Shift at position 1, S2M, D3Y and A12G. There was also a mutation in NP1-51 in the Lambda variant, P13F. None of these are shared with VOCs, and only D3Y, given the change in structure and charge may impact antibody binding. As with the VOCs, multiple mutation sites were found within NP164-215 (Figure 6F). Shared with VOCs were R203M (Kappa), R203K (Lambda), G204K (Lambda) and T205I (Eta). Additional mutations were P119L (Iota) and G214C (Lambda). The last two epitopes containing at least one variant with a mutation were NP232-269 and NP361-390, with one and two mutations respectively. These were M234I (Iota), T366I (Lambda) and D377Y (Kappa). Of these, D377Y results in a structural and charge change that may impact antibody binding.

### Shared Sequence Alignments Between SARS-CoV-2 Predicted Epitopes and Human Proteins

**FIGURE 7:**
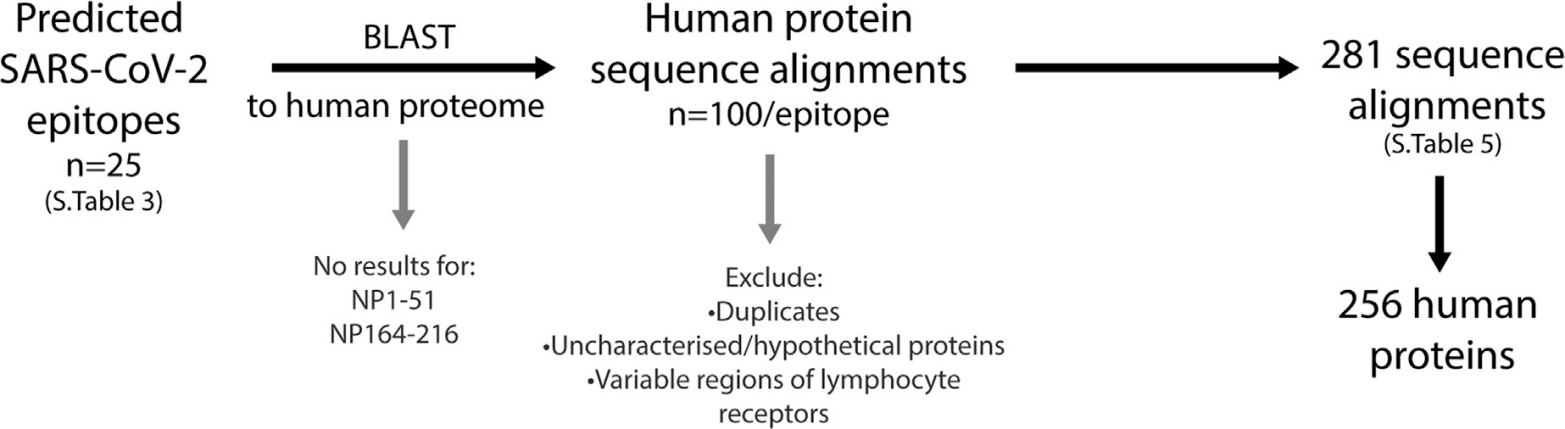
Workflow to identify human proteins that share sequence similarities with SARS-CoV-2 immunogenic regions. The predicted SARS-CoV-2 B cell epitopes were compared to the human proteome using the NCBI protein BLAST tool. Two epitopes, NP1-51 and NP164-216 had no sequence similarities to human proteins. The top 100 sequence alignments from the remaining 23 epitopes were narrowed by removing duplicates (alternative nomenclature/sequence IDs), uncharacterized/hypothetical proteins and the variable regions of lymphocyte receptors. This resulted in a final list of 281 sequence alignments that was comprised of 256 human proteins.

The similarities between the predicted SARS-CoV-2 B cell epitopes and the human proteome were identified using the NCBI protein-protein BLAST tool. Each of the 25 epitopes were compared, and final lists were narrowed to remove 1) the duplicates under alternative nomenclature or sequence ID 2) different isomer repeats 3) uncharacterised or hypothetical proteins and 4) variable regions of lymphocyte receptors (Figure 7; Supplementary Table S5). Of the 25 epitopes, two (NP1-51 and NP164-216) had no significant similarity when blasted to the nr database. Among the 23 other epitopes, a total of 281 alignments consisting of 256 self-proteins, were identified.

### Self-Proteins Share Disease Associations Which Relate to Symptoms Reported in COVID-19 Patients

**FIGURE 8:**
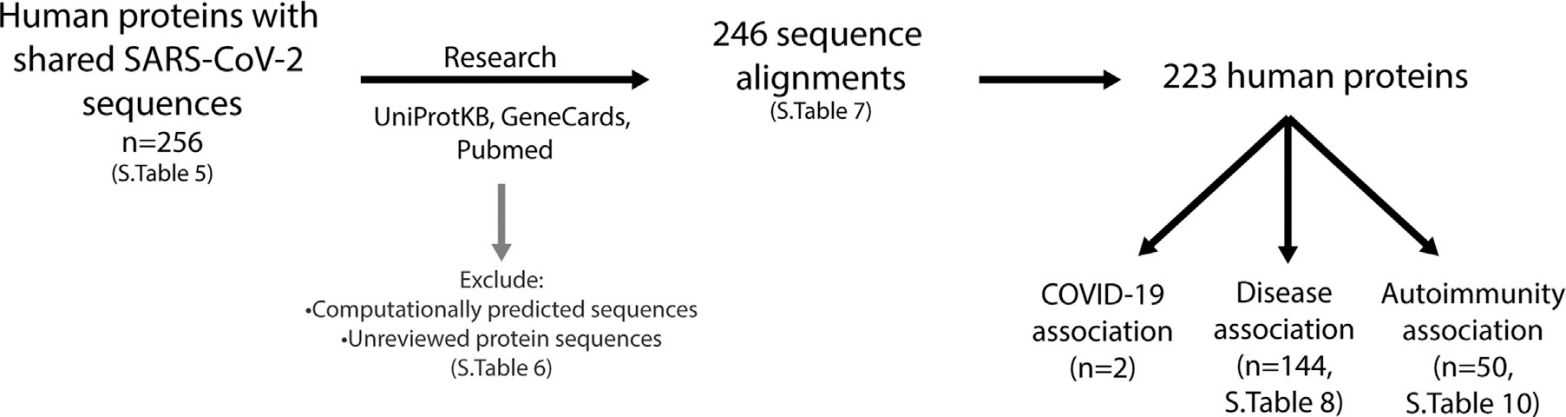
Investigation of human proteins and association with diseases. Using the UniProtKB, GeneCards and Pubmed online resources, research into expression location, protein function and association with diseases was performed on all proteins listed in Supplementary Table S5 (n = 256). This resulted in a further exclusion of proteins (listed in Supplementary Table S6), which were computationally predicted or unreviewed protein sequences, leaving 246 sequence alignments comprising of 223 proteins (Supplementary Table S7). Two proteins have been reported to be associated with COVID-19, 144 have an association with diseases (Supplementary Table S8) and 50 with autoimmunity (Supplementary Table S10).

For each of the human proteins identified that share similar sequences with the SARS-CoV-2 predicted epitopes (Supplementary Table S5), the function, association to diseases (including COVID-19) and the specific sequence alignment identified were investigated (Figure 8). In doing so, it was found that some of the alignments identified were in computationally predicted sequences or unreviewed proteins in the UniprotKB database. These proteins (Supplementary Table S6), were removed from those of interest, resulting in a total of 246 alignments, consisting of 223 self-proteins remaining (Supplementary Table S7).

While alignments identified within these remaining self-proteins did not overlap key functional domains of the human proteins, identified in the UniProtKB database, some of the proteins are described to be associated with a variety of diseases. Two of the human proteins identified have been reported in other SARS-CoV-2 related papers (Aishwarya et al., 2020; Vastrad et al., 2020). Ankryin B, which aligns to SP597-606, has been reported to be transcribed in lungs of COVID-19 patients, where it is not usually expressed (Aishwarya et al., 2020), and ZNF354C, aligning to NP361-390, is a transcription factor which interacts with genes that are differentially expressed in SARS-CoV-2 infected patients (Vastrad et al., 2020). Additionally, 144 of the self-proteins are reported to be associated with a range of other diseases (Supplementary Table S8). Among the identified diseases, we observed similarities for different forms of the same disease, for example types of retinitis pigmentosa or types of epilepsy. Some of these diseases share similarities with symptoms reported within COVID-19 patients such as cardiovascular diseases (atherosclerosis, cardiomyopathy, hypertension etc.) (Zhou et al., 2020b; Madjid et al., 2020); respiratory issues (airway hyper-responsiveness, inflammation) (Huang et al., 2020); neurological diseases (cerebellar ataxia, epilepsy) (Mithani et al., 2021; Povlow and Auerbach, 2021; Werner et al., 2021); and myopathy (Manzano et al., 2020; Versace et al., 2021). We also found that 42 of these 144 proteins had an association with various types of cancer.

**FIGURE 9:**
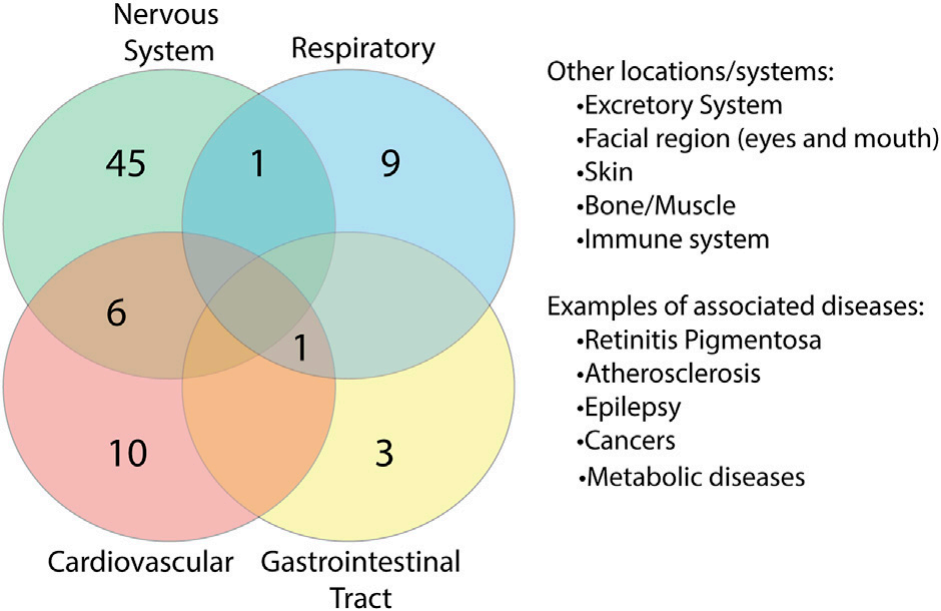
Overlap of proteins between body systems. Proteins found to be associated with diseases were grouped based on body system location of the diseases. Key systems with known complications in COVID-19 disease were found to have overlapping protein associations.

To identify whether there were any similarities or common location associations between the proteins, we grouped each of the proteins based on the body system/s they were found to be associated with (Supplementary Table S9). In doing so, we identified a range of overlap between proteins and systems (Figure 9). 52 proteins were found to be associated with the nervous system, six of which overlapped with the cardiovascular system, which is just under half of the cardiovascular-related proteins identified. Overlap could additionally be found with the respiratory system and gastrointestinal tract (GIT), systems associated with known COVID-19 complications. Additional locations/systems found to have protein associations included excretory system, facial region, skin, bone/muscle, thyroid, mitochondria/metabolic diseases and the immune system. Among the proteins associated with these, some showed overlap with other regions. This suggests that potential interruptions in some of these proteins could have multi-organ consequences, which may be associated with COVID-19.

### There is an Association Between Human Proteins, With Shared SARS-CoV-2 Sequences, and Autoimmunity

**FIGURE 10:**
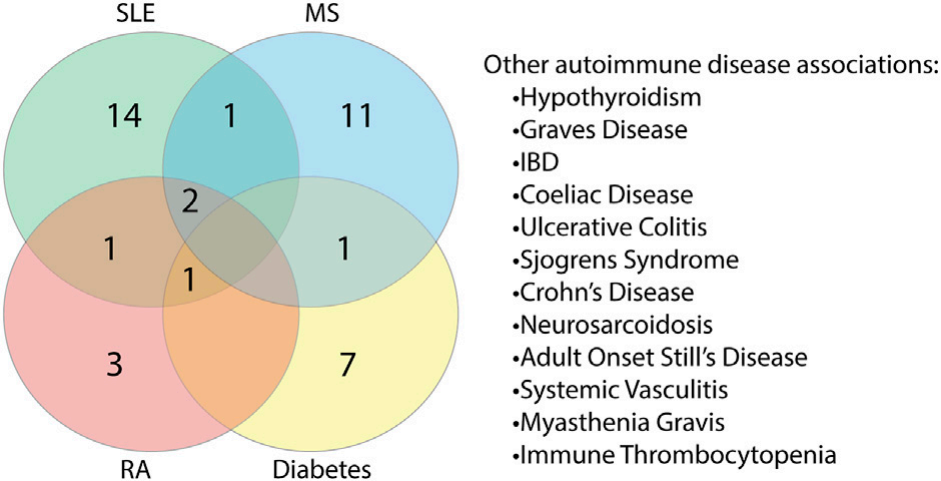
Proteins found to share alignments with SARS-CoV-2 epitopes have associations with various autoimmune diseases. Proteins associated with autoimmune diseases were grouped based on the specific disease or diseases (human and animal model combined) they are found to be associated with Systemic lupus erythematosus (SLE), multiple sclerosis (MS), rheumatoid arthritis (RA) and diabetes had the most protein associations. Proteins were also found to be associated with other autoimmune diseases to a lesser extent.

As reports of autoimmunity in COVID-19 continue to emerge (Bordet et al., 2020; Korem et al., 2020; Unsworth et al., 2020; Lui et al., 2021), of key interest was the association between the identified human proteins and whether they have a role in autoimmune diseases or are known autoantigens. Of the 223 human proteins, 50 were associated with autoimmune diseases, in both human and animal model settings (Supplementary Table S10). Among these 50 proteins, we found that some overlapped with multiple autoimmune diseases (Figure 10; Supplementary Table S11). Systemic lupus erythematosus (SLE) was found to have the most protein association, followed by multiple sclerosis (combined human and animal model, experimental autoimmune encephalomyelitis (EAE)). SLE shared the most overlap with other autoimmune diseases and some targets were shared across more than two autoimmune diseases. Many of the associations identified were due to gene single nucleotide polymorphisms (SNPs) and altered expression levels. However, eight of the proteins are known targets of autoantibodies and include key antibodies for assessing or diagnosing the associated diseases such as the myasthenia gravis autoantigen A-kinase anchor protein 12 (gravin), and histone 3, a nuclear target in SLE (Table 1). This suggests that the presence of some of these autoantibodies in COVID-19 patients without a history of autoimmune disease could be due to immune cross-reactivity.

**TABLE 1:** Identified human proteins which are known antigens of autoantibodies in autoimmune diseases.

SARS-CoV-2	Human protein	Autoimmune disease	References
SP 675-687	Serine/arginine-rich splicing factor 7	Multiple sclerosis	Ayoglu et al. (2016)
NP 361-390	RNA polymerase-associated protein RTF1 homolog	SLE and systemic vasculitis	Avila et al. (2008), Luo et al. (2019)
Orf3a 172-197	Golgin subfamily B member 1	RA, SLE and Sjögren’s syndrome	Rodríguez et al. (1982), Hong et al. (1992), Nozawa et al. (2004)
Orf3a 216-225	Tubby-related protein 3	SLE	Luo et al. (2019)
Orf3a 238-272	Centromere protein Q	SLE and systemic sclerosis	Song et al. (2013)
Orf3b 9-28	A-kinase anchor protein 12 (Gravin)	Myasthenia gravis	Gordon et al. (1992), Sasaki et al. (2001)
Orf7a 71-96	Alpha-Internexin	Type 1 diabetes, Hypothyroidism	Rajasalu et al. (2004)
Orf8 48-56	Histone H3.1	SLE	Sun et al. (2008), Didier et al. (2018)

### Potential Cross-Reactivity of Predicted SARS-CoV-2 B Cell Epitopes to Human Proteins

Short identical alignments (5-6aa) have been reported to be shared with SARS-CoV-2 and human proteins (Angileri et al., 2020a; Angileri et al., 2020b; Kanduc, 2020; Kanduc and Shoenfeld, 2020; Lucchese and Flöel, 2020; Marino Gammazza et al., 2020). Using the alignments identified from blasting predicted SARS-CoV-2 immune epitopes, the potential for immune cross-reactivity was explored. While point mutations in viruses are known to create antibody escape variants (Doud et al., 2018), this is not always the case, and not all mutations will affect antibody binding (Doud et al., 2018). Some antibodies are unaffected by conserved changes (Rodpothong and Auewarakul, 2012)**.** Therefore, to look at potential immune cross-reactivity, we applied criteria that not only relied on a minimum 6aa length, but took into account amino acid variations that had conserved charge and non-structural changes and therefore is less likely to impact antibody binding. Applying these unique criteria, a total of 136 alignments from both intra- and extra-cellular proteins were identified as potential cross-reactive targets (Supplementary Table S12). Although antibodies have a potential to cross-react with any of these proteins, they are more likely to bind and cross-react with extracellular proteins on intact cells. Using the UniProtKB database, research into the localization of each protein from Supplementary Table S12 of potential cross-reactive human proteins was performed. Those of further interest were proteins that are reported to be secreted into the extracellular domain or where the alignment region was reported to be extracellular. In doing this, a total of 11 proteins were identified (Table 2). In these 11 human proteins, the alignment identities to the SARS-CoV-2 epitopes ranged between 62 and 100%. Additionally, the SARS-CoV-2 spike and nucleoprotein epitopes with aligned human proteins were not found to have a mutation in any variant of concern or variant of interest.

**TABLE 2:** Extracellular human proteins with the potential for antibody cross-reactivity with SARS-CoV-2 immunogenic regions.

SARS-CoV-2				Human protein		
Epitope	Sequence	Predicted Score	Variants^a^	Protein	Alignment sequence^b^	Alignment Identities (%)
SP1256-1265	FDEDDSEPVL	>1.5	Alpha, Beta, Gamma, Delta, Eta, Iota, Kappa, Lambda	Bone morphogenetic protein 1 (BMP1)	E**EDDSEP**	86
NP115-127	TGPEAGLPYGANK	>1.5	Alpha, Beta, Gamma, Delta, Eta, Iota, Kappa, Lambda	CC chemokine STCP-1 (CCL22)	**EAG-PYGAN**	89
NP273-287	AFGRRGPEQTQGNFG	>2.0	Alpha, Beta, Gamma, Delta, Eta, Iota, Kappa, Lambda	Collagen alpha 3 (VI) chain (COL6A3)	**FGRRGP**	100
NP408-419	QQSMSSADSTQA	>1.5	Alpha, Beta, Gamma, Delta, Eta, Iota, Kappa, Lambda	Lysozyme like -1	**S**V**SSADST**E	78
				Lysozyme like -2	**S**V**SSADST**E	78
Orf3a 172-197	GDGTTSPISEHDYQIGGYTEKWESGV	0.63	NA	Mucin-12	**G**ES**TTSPIS**	78
Orf3a 216-225	STQLSTDTGV	0.6	NA	Tubby like protein 3	**S**S**Q**N**STDTG**I	80
Orf3b 9-28	SCCFSERFQNHNPQKEMATS	0.61	NA	Reelin	**SERFQN**	100
Orf7a 33-51	EPCSSGTYEGNSPFHPLAD	0.62	NA	Putative solute carrier organic anion transporter family member 1B7 (LST3)	**TY**D**GNSP**	86
Orf7a 71-96	VKHVYQLRARSVSPKLFIRQEEVQEL	0.6	NA	Lactase-phlorizin hydrolase	I**SP---**V**RQEEVQ**	62
				Alpha Internexin	FV**RQ**VHD**EEV**A**EL**	62

a Variants which contain no mutation.

b Bold letters indicate identical residues between human and SARS-CoV-2 alignments.

Next, we further explored these sequences in the human proteins through identifying whether they are potential epitopes within the self-protein sequence and their structural accessibility. Complete human protein sequences were obtained from the UniprotKB database and epitope mapping performed (Table 3). The alignments in seven of the 11 proteins were identified as epitopes, namely Bone morphogenetic protein 1, Lysozyme-like 1, Lysozyme-like 2, CCL22, COL6A3, tubby like protein 3 and alpha-internexin. Additionally, those of COL6A3, tubby like protein 3 and alpha-internexin were found within large sequences (greater than 50aa). For the proteins Reelin, LST-3 and lactase-phlorizin hydrolase, some, but not all, of the amino acids within the alignments were identified as potential epitopes. The final protein of interest, Mucin-12 did not yield any results when using the Bepipred Linear Epitope Prediction 2.0 algorithm, the algorithm used for the matching to SARS-CoV-2 protein, Orf3a. However, using version 1 of this prediction algorithm, the alignment partially sits within a predicted epitope. Given these epitopes were predicted, we searched IEDB to explore whether any of these B cell epitopes may have been previously validated, however none were. Despite this, most of these alignments do appear as potential epitopes within the human proteins, further suggesting a likelihood that antibody cross-reactivity may occur between SARS-CoV-2 and these targets.

**TABLE 3:** The identification of the human protein alignment as potential B cell epitope.

Human protein (UniProtKB ID)	Alignment location (aa)	Predicted epitope (start-end)	Prediction algorithm	Structured protein region (Y/N)
Bone morphogenetic protein 1 (BMP1, P13497)	34-40	Y (31-48)	Bepipred Linear Epitope Prediction V1.0	N
C-C motif chemokine 22 (CCL22, O00626)	23-30	Y (25-48)	Bepipred Linear Epitope Prediction V1.0	Y
Collagen alpha 3(VI) chain (COL6A3, P12111)	2200-2205	Y (2111-2374)	Bepipred Linear Epitope Prediction V1.0	N
Lysozyme-like 1 (Q6UWQ5-2)	16-24	Y (11-34)	Bepipred Linear Epitope Prediction V1.0	Y
Lysozyme-like 2 (Q7Z4W2-2)	16-24	Y (11-33)	Bepipred Linear Epitope Prediction V1.0	Y
Mucin-12 (Q9UKN1)	936-961, 2019-2044, 3576-3601, 4659-4684	NA	Bepipred Linear Epitope Prediction V2.0	N
Tubby Related protein 3 (O75386)	159-168	Y (5-187)	Bepipred Linear Epitope Prediction V2.0	N
Reelin (P78509)	1719-1724	N	Bepipred Linear Epitope Prediction V2.0	Y
Putative solute carrier organic anion transporter family member 1B7 (LST-3, G3V0H7)	394-400	N	Bepipred Linear Epitope Prediction V2.0	N
Lactase-phlorizin hydrolase (P09848)	1863-1869	N	Bepipred Linear Epitope Prediction V2.0	N
Alpha-Internexin (Q16352)	226-238	Y (154-241)	Bepipred Linear Epitope Prediction V2.0	Y

Y, Protein structure available or alignment is within a predicted epitope in the human protein; N, Protein structure not available or the full alignment could not be found within a predicted epitope; NA, epitope prediction results were not obtained.

**FIGURE 11:**
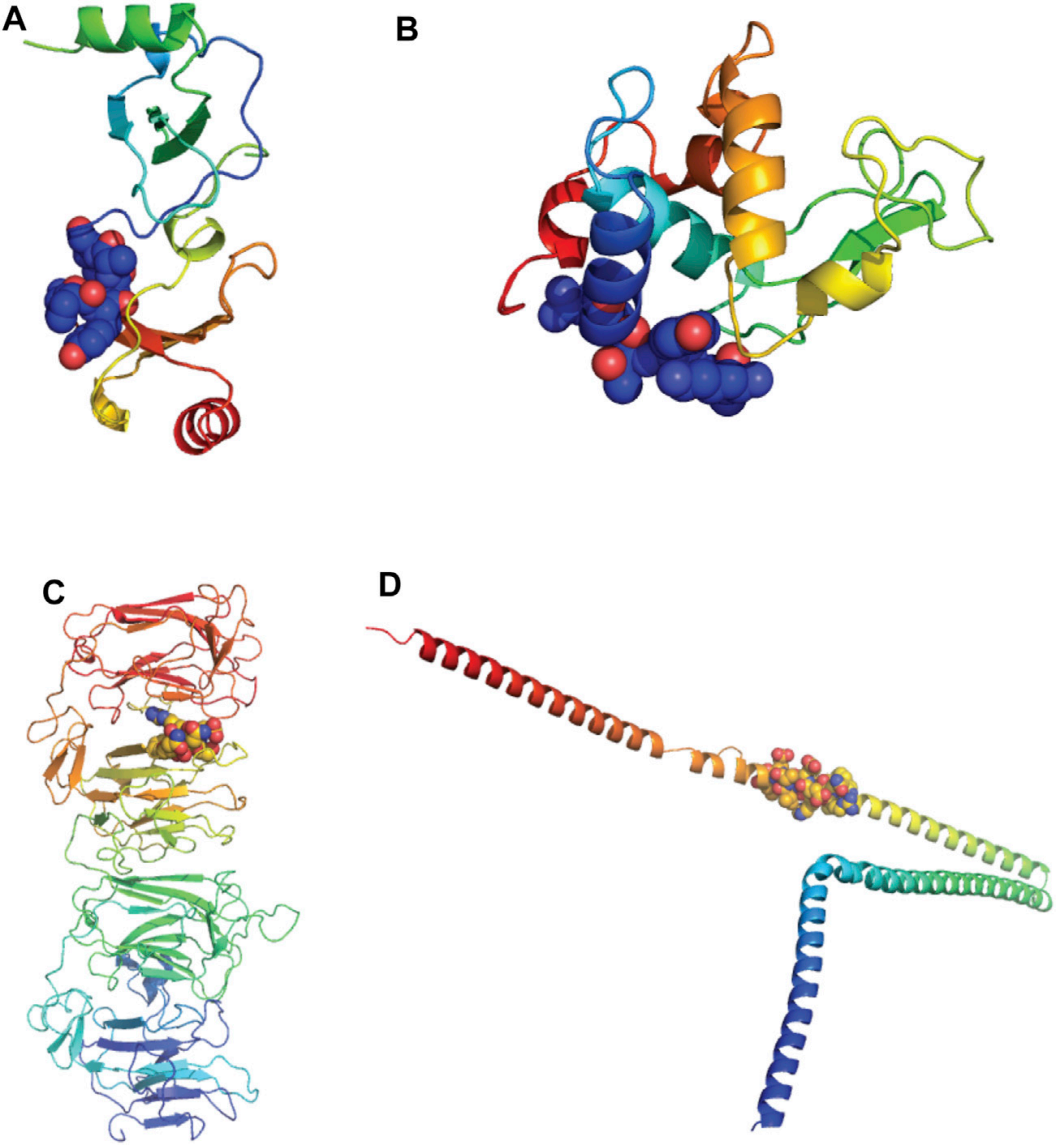
Alignments of extracellular proteins of interest mapped to protein structures. Homology models of human proteins of interest were downloaded from the SWISS-MODEL Repository and colored rainbow from N to C termini. The alignment motifs similar to SARS-CoV-2 B cell epitopes are highlighted *via* spheres (Bienert et al., 2017) **(A)** C-C motif chemokine 22 (CCL22). Model coverage aa25-91, alignment location aa23-30. **(B)** Lysozyme-like protein-1 and -2. Model coverage aa20-146, alignment location aa 16-24. **(C)** Reelin. Model coverage aa1251-1947, alignment location 1719-1724. **(D)** Alpha-internexin. Model coverage aa90-302, alignment location aa226-238.

Of the 11 extracellular proteins, five had homology model structures available which covered the alignment regions of interest: CCL22, Lysozyme-like 1, Lysozyme-like 2, Reelin and Alpha Internexin (Table 2). Each of these alignment motifs were mapped to the corresponding structures (Figure 11). In every case, the motifs of interest were found towards the protein surface, making them accessible to antibody binding and therefore the potential of cross-reactivity. Furthermore, for those proteins which did not have a structure readily available, all alignment motifs were predicted to reside in unstructured protein regions. These unstructured regions, usually unstructured loops or N-/C-terminal tails, are also readily available for antibody cross-reactivity.

### Thrombosis and Thrombocytopenia Syndrome Following COVID-19 Vaccination is Not due to Molecular Mimicry

Autoantibodies targeting PF4 have been implicated in the thrombosis and thrombocytopenia syndrome (TTS) induced in rare cases following vaccination with the ChAdOx1 nCoV-19 (AstraZeneca) or the Ad26.COV2.S (Johnson & Johnson) COVID-19 vaccines (Greinacher et al., 2021; Muir et al., 2021; Scully et al., 2021). Both these vaccines consist of adenovirus vectors encoding the spike protein of SARS-CoV-2 (Folegatti et al., 2020; Sadoff et al., 2021). To explore whether molecular mimicry is a potential mechanism causing the syndrome we used the blastp protein alignment tool to align the predicted spike epitopes to PF4 (UniprotKB: P02776). No similarity was found between any predicted epitope in the spike protein to sequences in the full-length PF4 protein. To further check if there may be similarity between the spike and PF4 outside the selected epitope regions, the complete spike protein sequence was compared to PF4, which resulted in no similarity results. PF4 interacts with a variant of CXCR3 (CXCR3B) (Lasagni et al., 2003), the receptor of a number of key chemokines (such as CXCL10, a pro-inflammatory cytokine important for chemotaxis and the activation of peripheral immune cells (Lasagni et al., 2003)). We therefore explored whether the SARS-CoV-2 spike epitopes were similar to CXCR3B and thus potentially involved with interrupting PF4-CXCR3B interactions. Spike epitopes were blasted to the CXCR3-B UniprotKB sequence (P49682-2), and no similarities were identified. Finally, as deficiency of ADAMTS13, a metalloprotease, has been implicated in patients with ITP, spike epitopes were blasted to the ADAMTS13 UniprotKB sequence (Q76LX8), where no sequence similarity was found. This suggests that molecular mimicry is unlikely to be the cause of the described cases of TTS.

## Discussion

Using *in silico* immunoinformatic tools, potential B cell immunogenic epitopes in the SARS-CoV-2 proteome were predicted and further used to compare to global variants as well as explore the similarity to human proteins. In doing so, we identified eight structural epitopes containing mutations in at least one strain within these immunogenic regions. When comparing the epitopes to the human proteome, a variety of human proteins were identified to share sequences similar to SARS-CoV-2 proteins. Many of the identified human proteins were found to be associated with diseases, some of have which been reported to be related to COVID-19 symptoms and complications. Additionally, we show associations of these proteins to autoimmune diseases, such as SLE and MS. We further identified sequence similarities between SARS-CoV-2 immunogenic regions and human proteins which are localized in the extracellular region. These similarities and potential ease of access to circulating antibodies suggests the potential damaging cross-reactivity that can perpetuate a pathological condition. Finally, we analyzed and found that molecular mimicry may not be the mechanism for the thrombosis and thrombocytopenia syndrome occurring following vaccination with the AstraZeneca and Johnson & Johnson COVID-19 vaccines.

To the best of our knowledge, this is the first study to predict B cell epitopes and compare to the highlighted VOCs and VOIs known to escape the immune response and be more infectious. However, a recent study aligned 10,664 SARS-CoV-2 genomes, to identify conserved regions and predicted both B and T cell epitopes specifically within these regions (Ghosh et al., 2021). Of their highlighted B cell epitopes, only our single predicted membrane epitope crossed over. This may be due to different epitope prediction algorithms used or single mutations in these genomes eliminating regions of interest due to not being conserved. While this method, may help in the design of epitope-based synthetic vaccines, due to targeting conserved regions, it does not indicate the immunogenic regions of the full proteins and how mutations may impact immune responses. Typically, the mutations reported in the VOCs and VOIs are those that lie in the spike protein due to its key role in infection and pathogenesis (Shang et al., 2020). D614G was one of the earliest mutations in variants that emerged as more infectious than the initial SARS-CoV-2 variant and became globally dominant (Korber et al., 2020). Each of the VOCs contain this mutation along with various others. The Alpha strain contains a N501Y mutation in the ACE2 receptor binding domain (RBD) (Garcia-Beltran et al., 2021). Examples of other mutations characteristic to the different strains used in the present study include K417N/T and E484K (Garcia-Beltran et al., 2021), and P681H, L425R, P681R, E484Q, among others (COVID-19 Weekly Epidemiol, 2021). Of these, one mutation of interest in the spike protein, P681H/R, is located next to the furin cleavage site which is important for invading host cells (Papa et al., 2021; Peacock et al., 2021). Specifically, P681R is of interest as it is found within the Delta variant. Following the wave of devastation in India, with its increased transmissibility, the Delta variant has become the dominant variant in multiple countries (Bolze et al., 2021; Mishra et al., 2021; Sheikh et al., 2021). An early study suggests mutation P681R is associated with the increased viral pathogenicity (Saito et al., 2021). This mutation site can be identified within the predicted epitope SP675-687. This epitope is the only spike predicted epitope that contained at least one mutation in multiple VOCs (Alpha, Beta and Delta) and VOIs (Eta and Kappa) but is also the only predicted spike epitope not overlapping a validated epitope. Despite this, there may still be antibody responses to this epitope in the population, and therefore the mutations within the SP675-687, such as P681R, may be impacting antibody binding due to structure and charge changes that could be impacting a functional role of these antibodies. Among the other predicted spike epitopes only SP249-261 contained at least one mutation in VOIs, Iota and Lambda. With no mutations identified in the other three spike epitopes, this suggests that although found overlapping validated regions, these antibodies may not play a role in viral neutralization. We additionally found mutations in at least one variant in six of the nine predicted nucleoprotein epitopes. NP164-216 was found to contain multiple mutations among all VOCs and VOIs examined, all of which were around the same region (position 199-205 and 214). It has recently been reported that high anti-NP responses may be associated with poorer outcomes in COVID-19 (Batra et al., 2021). With more severe disease associated with these variants, it raises questions of whether the mutations in these epitope regions increase antibody binding, rather than decrease binding, or whether the different strains have unique epitope regions.

COVID-19 is associated with a series of multi-organ complications (Huang et al., 2020; Zaim et al., 2020). Many of the human proteins identified in this study, that share amino acid sequence similarities with the SARS-CoV-2 virus, play key roles in cellular functions, which if interrupted may result in altered cell function and therefore pathology. We found that clusters of proteins could be grouped based on their relationship to similar diseases and overlap to multiple body systems, some of which have been implicated in COVID-19 pathology, including respiratory, cardiovascular, gastrointestinal tract and nervous systems. Some of the broad examples of such diseases include epilepsy, cardiomyopathy and cerebellar ataxia, all of which have been reported in COVID-19 patients (Siripanthong et al., 2020; Mithani et al., 2021; Povlow and Auerbach, 2021; Werner et al., 2021). However, some of the diseases associated with the similar proteins may not result in a complication but instead confer a higher risk. Alzheimer’s Disease, macular degeneration and cardiovascular diseases were all diseases identified with proteins that shared sequence similarities to SARS-CoV-2 capable of making them the targets of autoantibodies. Pre-existing diagnosis for each of these have been found to predict higher risk of infection and greater severity and risks in COVID-19 (Chen et al., 2020; Huang et al., 2020; Ramlall et al., 2020; Yu et al., 2021). Many of the proteins identified to be associated with disease are intracellular and are therefore less likely to be immune targets. However, as the SARS-CoV-2 virus is an intracellular pathogen, the sequence similarities could alternatively have an impact on cellular functions which may result in the observed pathologies, independently of having the potential to be recognized by antibodies.

Autoantibody (AAb) targeted proteins found within a range of autoimmune diseases (SLE, Myasthenia Gravis, T1D etc.) were found to share similar sequences to some of the new predicted SARS-CoV-2 epitopes, as well as known SARS-CoV-2 epitopes (Shrock et al., 2020; Wang et al., 2020). Studies have shown that within COVID-19 patients, known AAbs associated with autoimmune diseases, including but not limited to, anti-cardiolipin, anti-SSA/Ro and anti-nuclear antibody (Zhou et al., 2020a; Vlachoyiannopoulos et al., 2020) are increased, indicating a breaking of immune tolerance (the mechanisms which regulate responses and ensure immune cells do not attack self). As identified in the present study, histone H3 shares an identical 6aa sequence with the SARS-CoV-2 Orf8 protein, which additionally sits in a region identified as an epitope in COVID-19 patients (Wang et al., 2020), indicating the possibility of immune cross-reactivity. Gravin, the myasthenia gravis autoantigen, was also found to have sequence similarity with the Orf3b viral protein. While, to our knowledge, anti-gravin AAbs have not been reported in COVID-19 patients, there has been a case report of post-COVID-19 infection onset of myasthenia gravis (Huber et al., 2020). With several viral infections being associated with autoimmune diseases, these data support the possibility that the COVID-19 pandemic will lead to an increase in autoimmune diseases. Anti-La and anti-Jo-1, typically associated with SLE/Sjogren’s Disease and inflammatory myopathies respectively, have been identified in children with COVID-19 Multisystem Inflammatory Syndrome (MIS-C) (Consiglio et al., 2020; Gruber et al., 2020). However, it is not only AAbs with known associations to autoimmune diseases found in COVID-19 patients (Consiglio et al., 2020; Gruber et al., 2020). AAbs to a range of tissue specific and immune related mediators have been identified in MIS-C patients. Additionally, increased levels of anti-interferon (IFN) antibodies have been reported to be associated with more severe COVID-19 disease (Bastard et al., 2020). This shows that it is not only important to understand the targets associated with autoimmune diseases, but that any similarity or recognition of self may contribute to additional COVID-19 pathology and/or greater disease severity.

The majority of proteins found to fit within our distinct criteria for the potential of cross-reactivity were intracellular, with only 11 found to be extracellular. By applying these criteria, two proteins associated as antigens in autoimmune diseases, Tubby-related protein 3 and Alpha-internexin, were also identified as potential cross-reactive proteins. We additionally found that tubby-related protein 3, as well as two other potential cross-reactive targets, bone morphogenetic protein 1 and Mucin-12, have alignments similar to SARS-CoV-2 epitopes which overlap with *in vivo* validated epitopes (Li et al., 2020; Shrock et al., 2020; Wang et al., 2020), potentially indicating an opportunity for cross-reactivity, especially as these targets are extracellular. Additionally, as seen in autoimmune diseases, many autoantibodies to various intracellular targets are identified (Suurmond and Diamond, 2015). During COVID-19 infection, the release of intracellular proteins may be playing a role in breaking immune tolerance, allowing for the potential cross-reactivity of SARS-CoV-2 antibodies to self, or the increase of AAbs. This may be perpetuated through the recognition of the regions that are extracellular and therefore more likely to be visible to circulating antibodies. Interestingly, the SARS-CoV-2 spike and nucleoprotein alignments with human protein matches conforming to our criteria, are ones that do not contain mutations across different global variants, suggesting the potential for cross-reactivity to these proteins irrespective of the virus variant. Furthermore, according to the IEDB, there are several confirmed discontinuous B cell epitopes within the spike protein, which may also further expand the number of cross-reactive epitope targets to human proteins to be explored in future studies.

Other studies have reported sequence similarities between SARS-CoV-2 and human proteins (Angileri et al., 2020a; Angileri et al., 2020b; Ehrenfeld et al., 2020; Kanduc, 2020; Kanduc and Shoenfeld, 2020; Lucchese and Flöel, 2020; Lyons-Weiler, 2020; Marino Gammazza et al., 2020), the majority through identifying identical 5 or 6 amino acid segments found within immunogenic regions (Angileri et al., 2020a; Angileri et al., 2020b; Ehrenfeld et al., 2020; Kanduc, 2020; Kanduc and Shoenfeld, 2020; Lucchese and Flöel, 2020; Marino Gammazza et al., 2020). Apart from the study of Kanduc (2020), who formed overlapping hexamers (offset by one residue) from SARS-CoV-2 sequences that are identical in validated SARS-CoV-1 epitopes, these studies began by aligning overlapping pentamers/hexamers to human proteins to identify identical sequences (Angileri et al., 2020a; Angileri et al., 2020b; Kanduc and Shoenfeld, 2020; Lucchese and Flöel, 2020; Marino Gammazza et al., 2020). These identical alignments were then determined if they were found in immunogenic regions. In contrast, Lyons-Weiler (2020) predicted immunogenic SARS-CoV-2 epitopes which were then compared to human proteins. In the present study, we used a similar approach to Lyons-Weiler (2020) by identifying sequences in key SARS-CoV-2 proteins with higher predicted immunogenic regions. However, the comparison between these SARS-CoV-2 sequences and human proteins was further expanded, in contrast to the other reports (Angileri et al., 2020a; Angileri et al., 2020b; Ehrenfeld et al., 2020; Kanduc, 2020; Kanduc and Shoenfeld, 2020; Lucchese and Flöel, 2020; Marino Gammazza et al., 2020), as we did not only rely on short identical sequences. Instead, to explore potential immune cross-reactivity, we applied a novel combination of criteria, which considered the ability of antibodies to potentially recognize amino acid differences, as well as the localization of the alignments for antibody accessibility. Although our criteria were unique compared to the previous studies, we’d expect some crossover with those who report identical hexamers (Angileri et al., 2020b; Ehrenfeld et al., 2020; Kanduc, 2020; Lucchese and Flöel, 2020; Marino Gammazza et al., 2020), and had a similar start methodology (Lyons-Weiler, 2020)**.** However, to the best of our knowledge only five previously reported proteins were also found in our potential targets: Hermansky-Pudlak Syndrome I protein, Unconventional myosin XVI, Transmembrane Protein KIAA1109 (Ehrenfeld et al., 2020), Ankyrin repeat and sterile alpha motif domain containing 1A (Lyons-Weiler, 2020) and CLOCK (Kanduc, 2020). Each of these proteins reported the same alignment to the same SARS-CoV-2 protein, except for CLOCK which was reported with a different hexamer alignment suggesting some proteins may have multiple shared sites with SARS-CoV-2. Other similar findings may not have been identified as these reports focus only on human proteins of a certain function or location (e.g. molecular chaperones or adaptive immune system proteins), some of which were found to match SARS-CoV-2 proteins not studied in the present study (e.g. orf1ab). Differences in findings may also be due to different *in silico* tools used for the comparison of SARS-CoV-2 and human proteins, where we used the NCBI blastp tool while others used the Pir Peptide Match program (Kanduc, 2020; Kanduc and Shoenfeld, 2020; Marino Gammazza et al., 2020). It was found that the Pir Peptide Match program preferred short sequences (∼6aa length) to identify the similar human proteins but could not obtain the same results when using the full-length epitopes (>10aa long). Whereas the NCBI blastp tool allowed us to apply criteria that not only relied on identical sequences but considered how similar charge and structure between amino acid variations may not impact antibody binding and therefore explore sequence similarity between the full length of the predicted epitopes and human proteins. Additionally, not all pentamers or hexamers from the SARS-CoV-2 proteins, reported to align with human proteins, can be found within our predicted epitopes, further explaining differences in the human proteins identified. In some cases, this may be due to different prediction algorithms used, or the sequences were part of prediction epitopes that did not fit our specific epitope criteria. One sequence, SRSSSR, found within the nucleoprotein, has been highlighted in multiple reports as having a shared alignment with human proteins (Angileri et al., 2020b; Kanduc, 2020). This hexamer can be found within our predicted epitope NP164-216, however this was one of the epitopes that obtained no significant blast results. This example demonstrates how there can be major differences in outputs between different bioinformatic methods.

Computational methods, such as epitope mapping and blasting are useful techniques. They can allow narrowing of questions and potential proteins of interest in hypotheses before doing experimental studies. However, while bioinformatics studies can narrow these lists, the current study is limited to predictions alone and ultimately need to be confirmed experimentally. Since the initial epitope predictions were performed, new literature emerged to identify new SARS-CoV-2 B cell epitopes *in vitro* that were also identified by our approach which have not been reported previously (Amrun et al., 2020; Poh et al., 2020; Shrock et al., 2020; Wang et al., 2020; Yi et al., 2020; Yoshida et al., 2021). Several of our predicted epitopes, including: Spike 249-261, 597-606, 805-816, 1256-1265; NP 164-216, 232-269, 361-390; Orf3a 172-197, 216-225, Orf8 23-45, 48-56 overlap with the new epitopes highlighted in these reports. Regions within spike 553-579 and 806-835 have been identified across several studies as immunodominant regions (Amrun et al., 2020; Poh et al., 2020; Yi et al., 2020), whereas other epitopes show differing ranges of reactivity (Shrock et al., 2020). This indicates that, although not immunodominant or reported in the literature, these predicted epitopes may still be real, especially as individual immune responses are polymorphic. This is important to consider when mapping potential cross-reactivities between SARS-CoV-2 and self-antigens, as there may still be protein cross-reactivities to identify due to host or virus polymorphisms. Additionally, to allow for direct sequence similarities between virus epitopes and human proteins, only linear B cell epitopes were explored in the present study. However, B cell epitopes can be composed of multiple discontinuous segments (Rubinstein et al., 2008). As more protein structures covering the full-length SARS-CoV-2 proteins are identified, performing discontinuous epitope analysis may further uncover other potential human proteins that may become targets of antibodies through cross-reactivity. Furthermore, T cells may also cross-react with self-protein T cell epitopes through mechanisms such as bystander activation (Smatti et al., 2019). A future study looking specifically at T cell epitopes and cross-reactivity to self may provide further evidence of the immune system’s role in pathology.

For the first time, to our knowledge, we further explored and identified that some of the alignments in self-proteins predicted to be cross-reactive with SARS-CoV-2 alignments are potential epitopes within their own complete sequence. Additionally, where possible, we applied structural analysis to further explore the potential for cross-reactivity to the human proteins. In doing so, we identified the motif alignments were towards the surface of the protein, or in unstructured regions, and therefore would provide ready access for antibody binding. As crystal structures become available for the human proteins of interest, both full coverage of those mapped, as well as the remaining proteins, it would be interesting to further apply this method. However, the Bepipred Linear Prediction Algorithm 2.0 derives its epitope predictions from crystal structures (Jespersen et al., 2017). It is therefore likely that the Orf proteins and the human cross-reactive alignments predicted to be epitopes, are more likely to be found on the surface where antibodies have easier access.

The mechanism behind the thrombotic events occurring in some people following vaccination with the AstraZeneca and Johnson & Johnson adenovirus vectored vaccines is not known (Scully et al., 2021). Our findings suggest that molecular mimicry between the SARS-CoV-2 spike protein and proteins implicated in TTS and ITP, including PF4 and ADAMTS13, is unlikely to be the cause of these events. The vaccine induced thrombosis thrombocytopenia syndrome has been reported to be similar to autoimmune heparin-induced thrombocytopenia (aHIT) (Schultz et al., 2021), and therefore similar mechanisms may be involved. In aHIT, it has been suggested that structural changes in PF4 may be involved (Greinacher et al., 2017). Anti-PF4 antibodies are part of the diagnostic criteria for TTS (Greinacher et al., 2021). It has been hypothesized that free DNA in the vaccines may be a possible trigger of the anti-PF4 antibodies (Greinacher et al., 2021). However, further research into the formation of these anti-PF4 autoantibodies and the causes behind TTS following COVID-19 vaccination is required to identify potential interventions to prevent these events.

In the present study, linear B cell epitopes in SARS-CoV-2 proteins were predicted and compared to human proteins, identifying a number of new targets for potentially cross-reactive autoantibodies. Since many of the predicted epitopes reported in this study overlap with ones validated in COVID-19 patients, future studies may synthesize peptide sequences from the SARS-CoV-2 epitopes and the equivalent human sequences to perform *in vitro* analysis for validation.

## Data Availability

The original contributions presented in the study are included in the article/Supplementary Material, further inquiries can be directed to the corresponding author.
