# Supplementary material for: Predicted B Cell Epitopes Highlight the Potential for COVID-19 to Drive Self-Reactive Immunity
Source: Front Bioinform. 2021 Aug 19;1:709533. doi: 10.3389/fbinf.2021.709533 (PMC9581003; doi:10.3389/fbinf.2021.709533)
Supplement: Supplementary file 2 [file DataSheet1.docx]

Supplementary Material

# Supplementary Figures

**A**

**
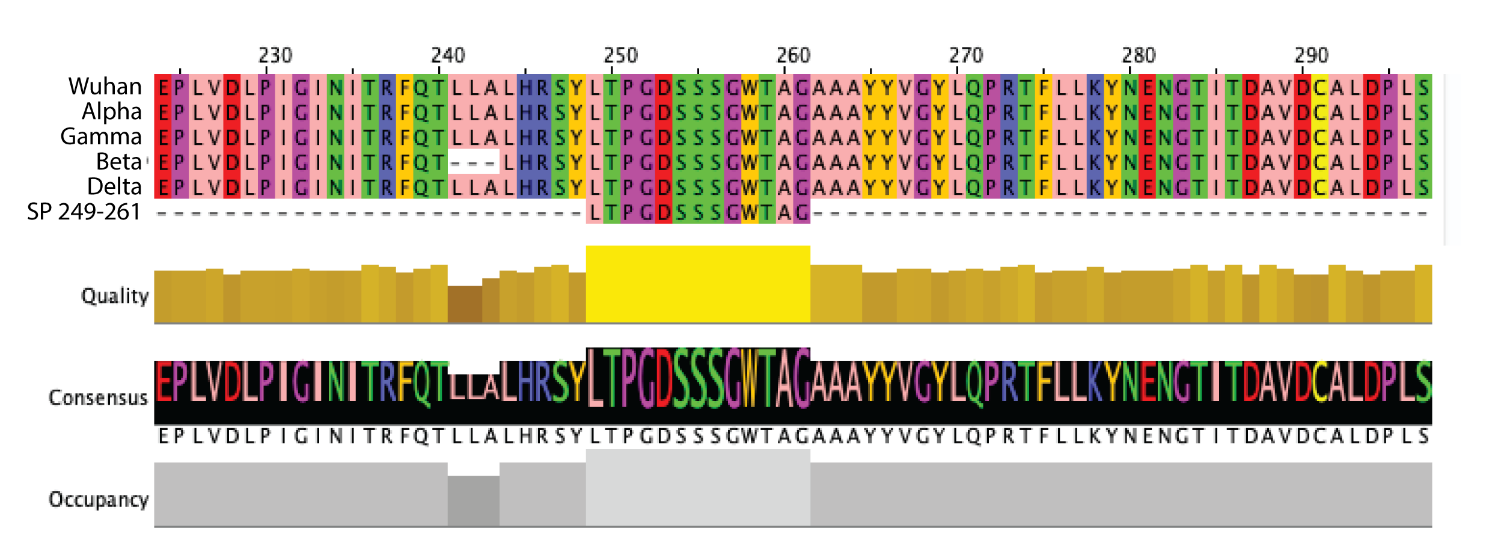
**

**B**

**
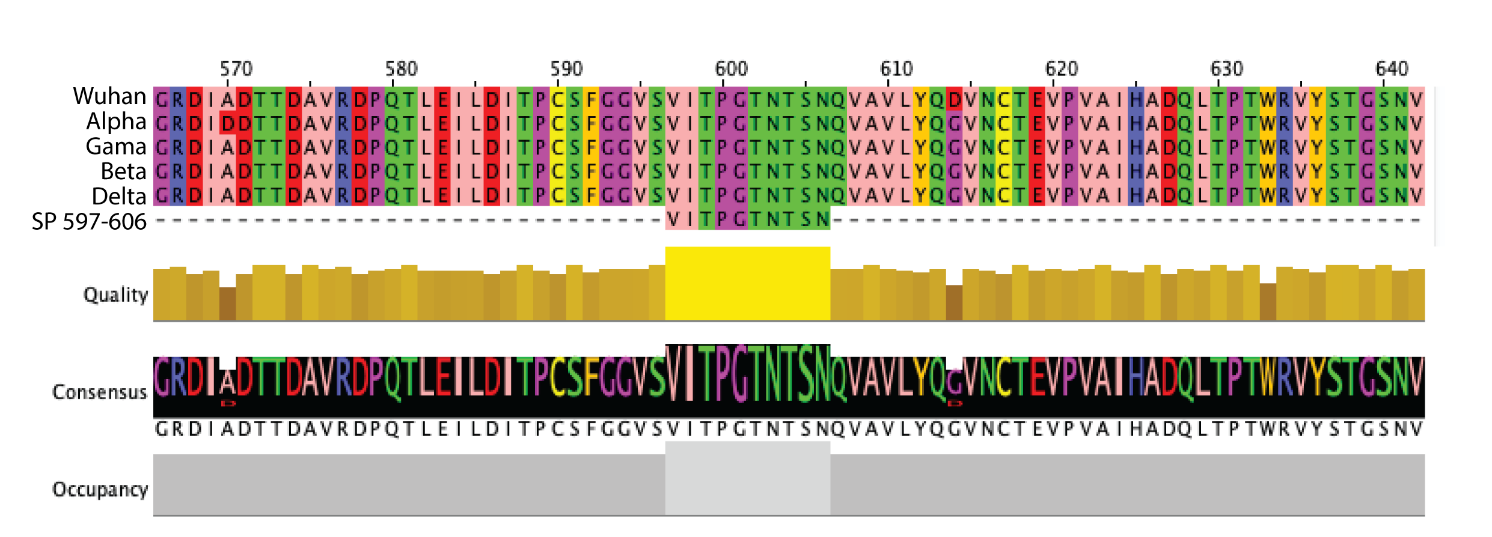
**

**C**

**
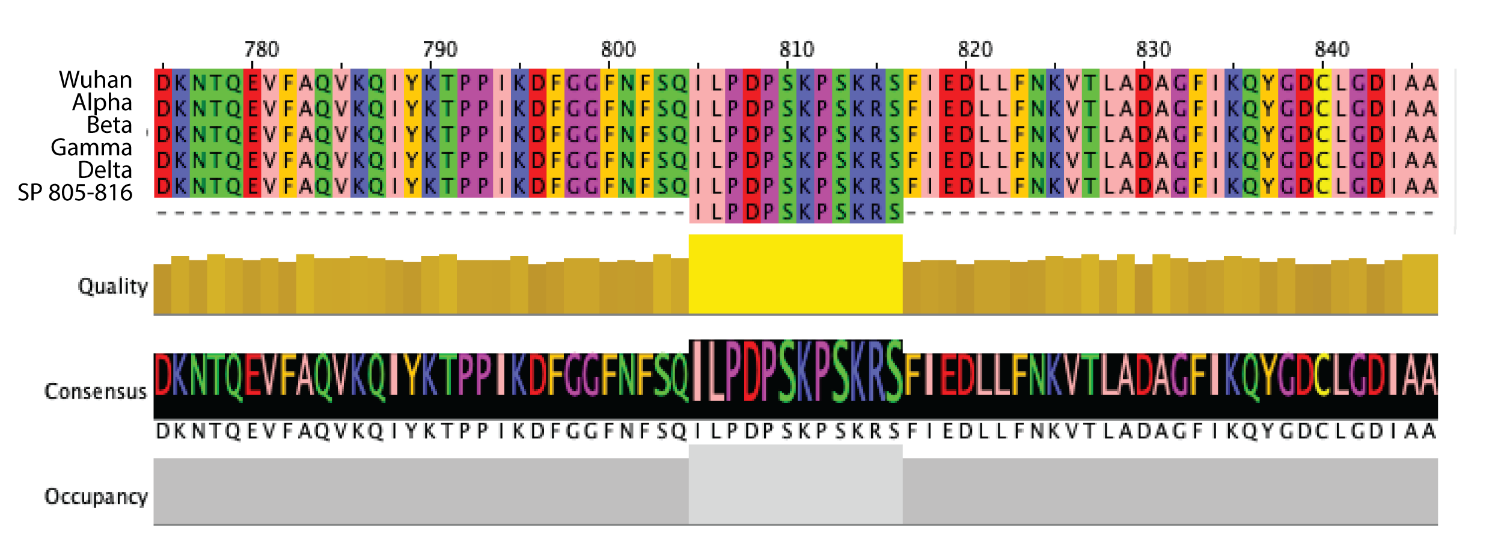
**

**D**

**
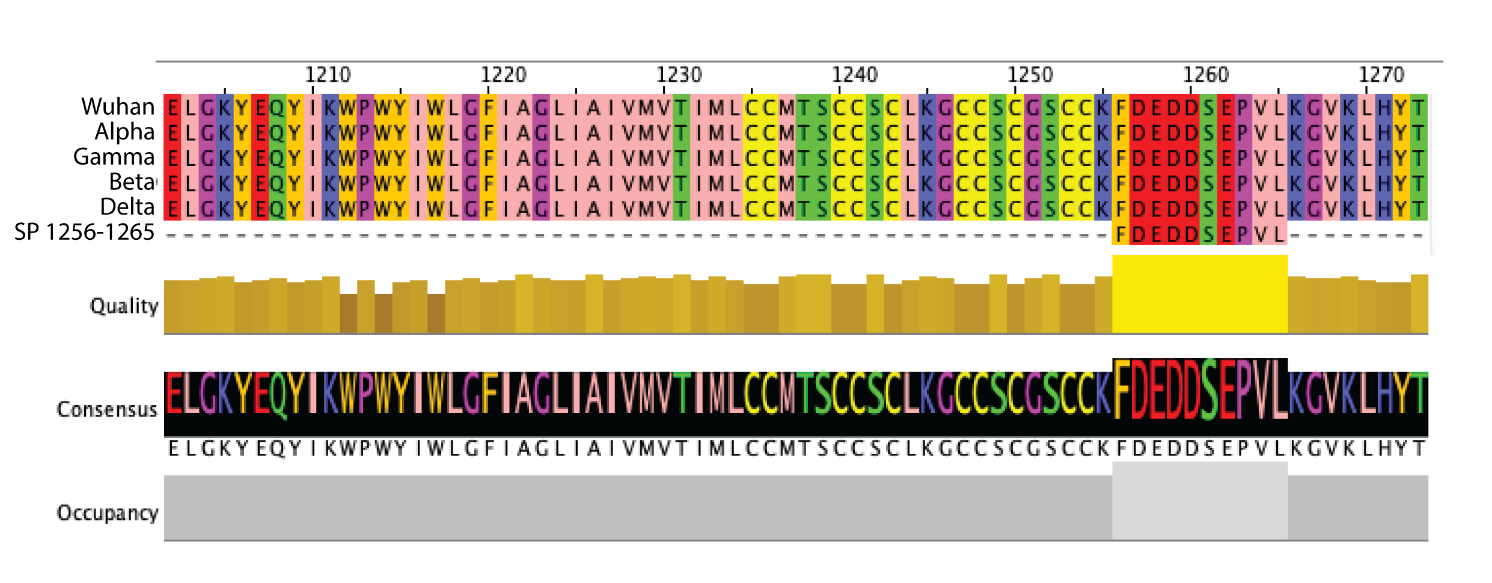
**

**Supplementary Figure 1.** Comparison of predicted spike epitopes, from original Wuhan strain to SARS-CoV-2 variants of concern: Alpha, Beta, Gamma and Delta. Quality refers to the alignment quality based on blosum2 algorithm scores, Consensus indicates the abundance of the amino acids present in a particular position and Occupancy is the number of aligned positions. (A) SP249-261 (B) SP597-606 (C) SP805-816 (D) SP1256-1265.

**A**


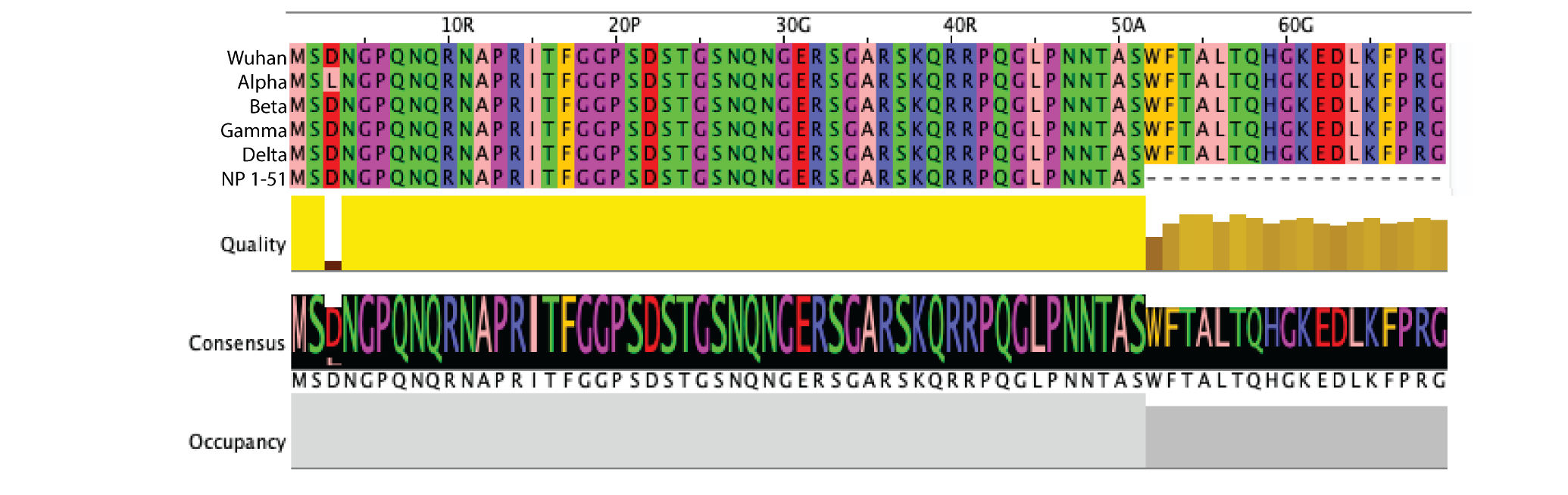


**B**

**
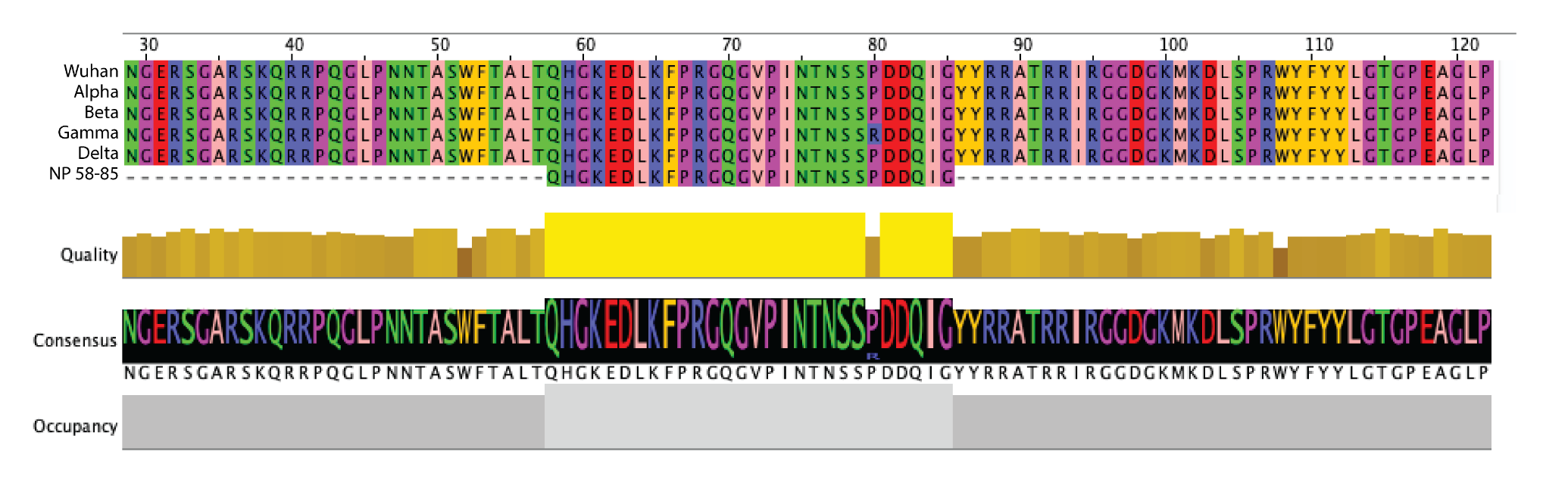
**

**C**

**
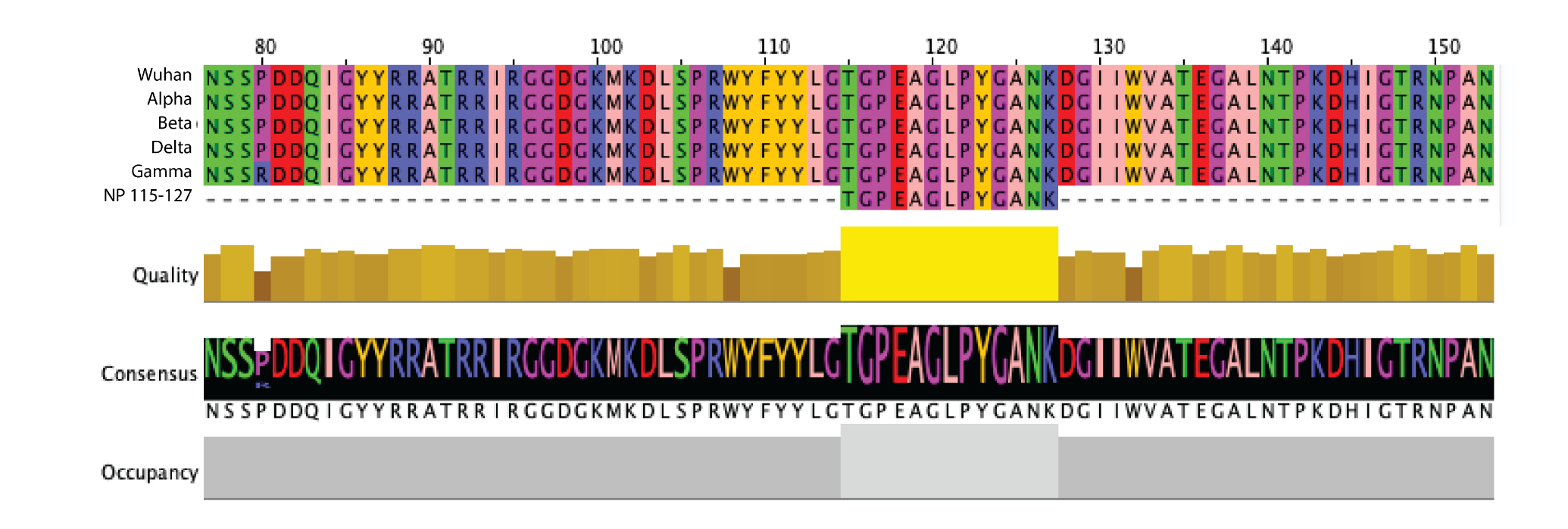
**

**D**

**
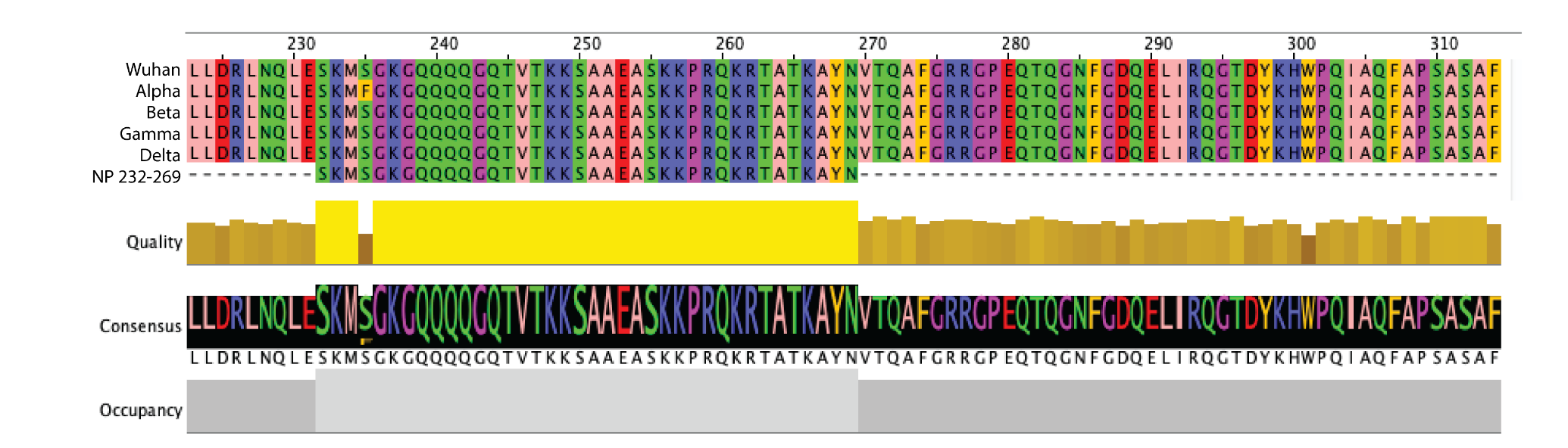
**

**E**

**
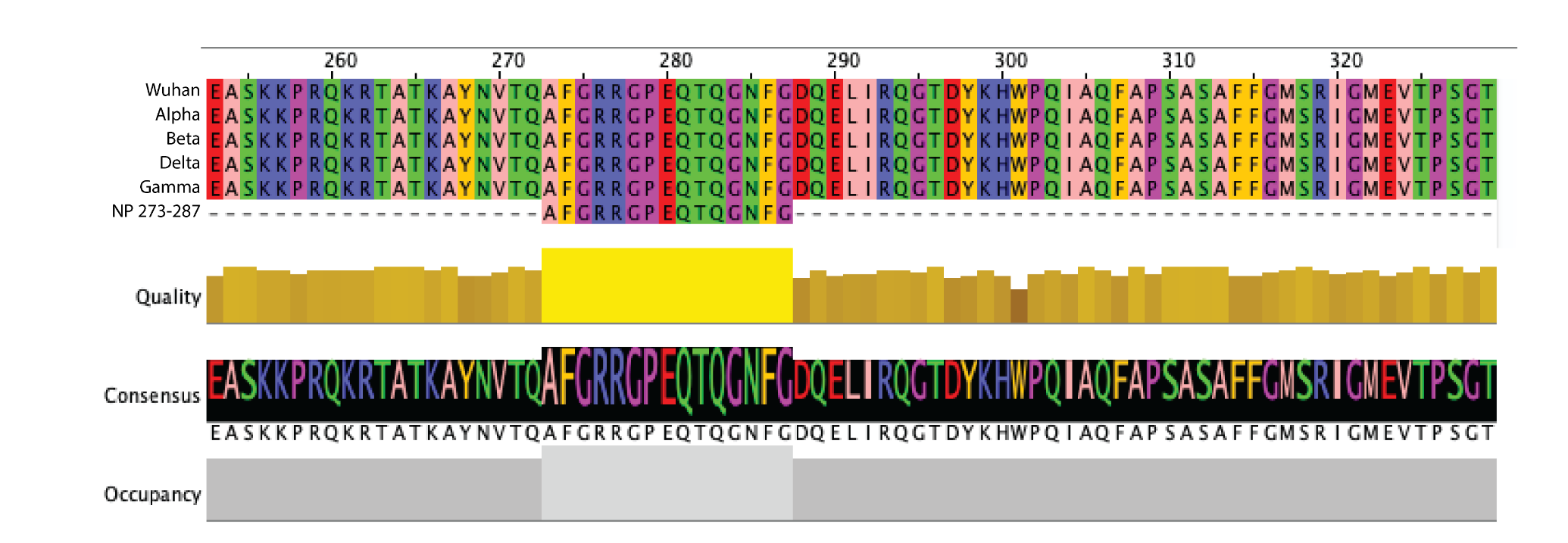
**

**F**

**
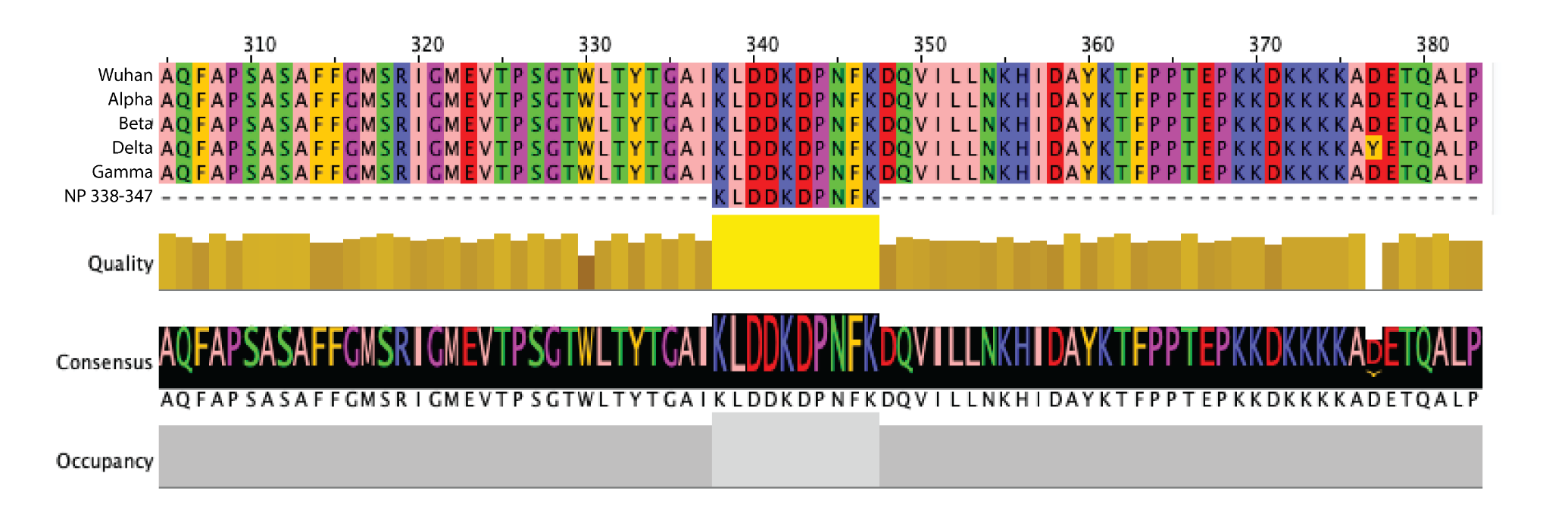
**

**G**

**
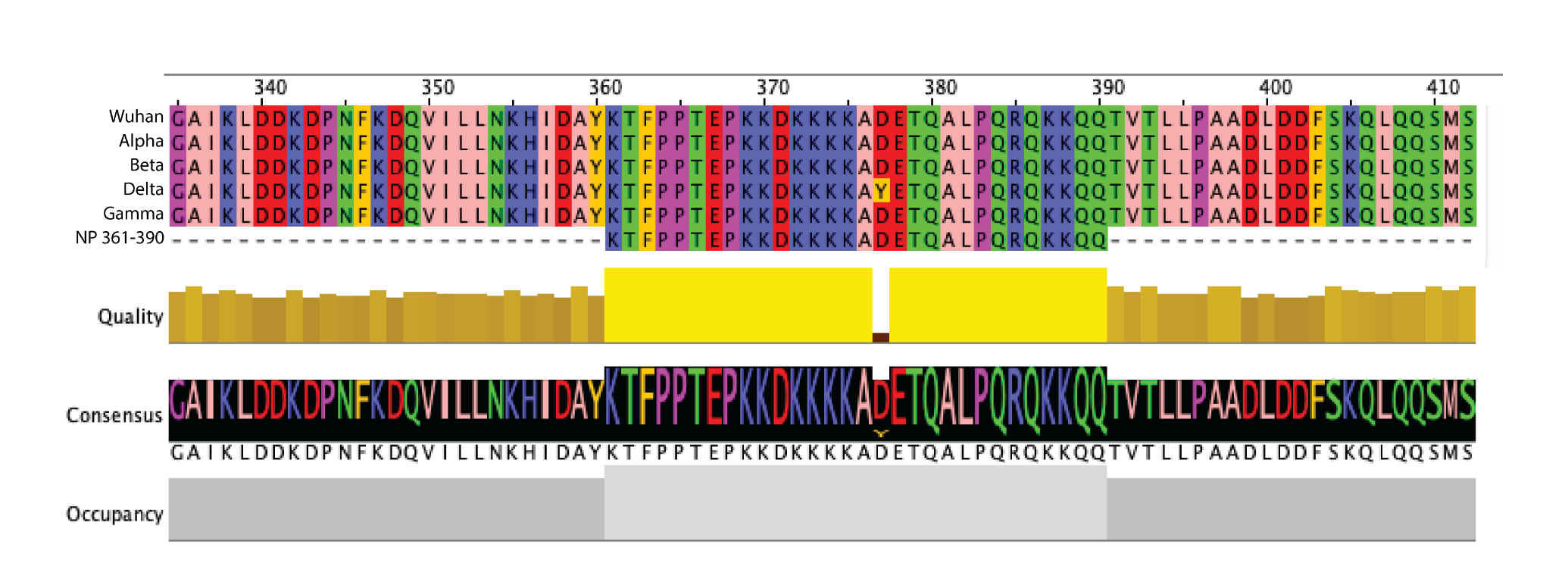
**

**H**

**
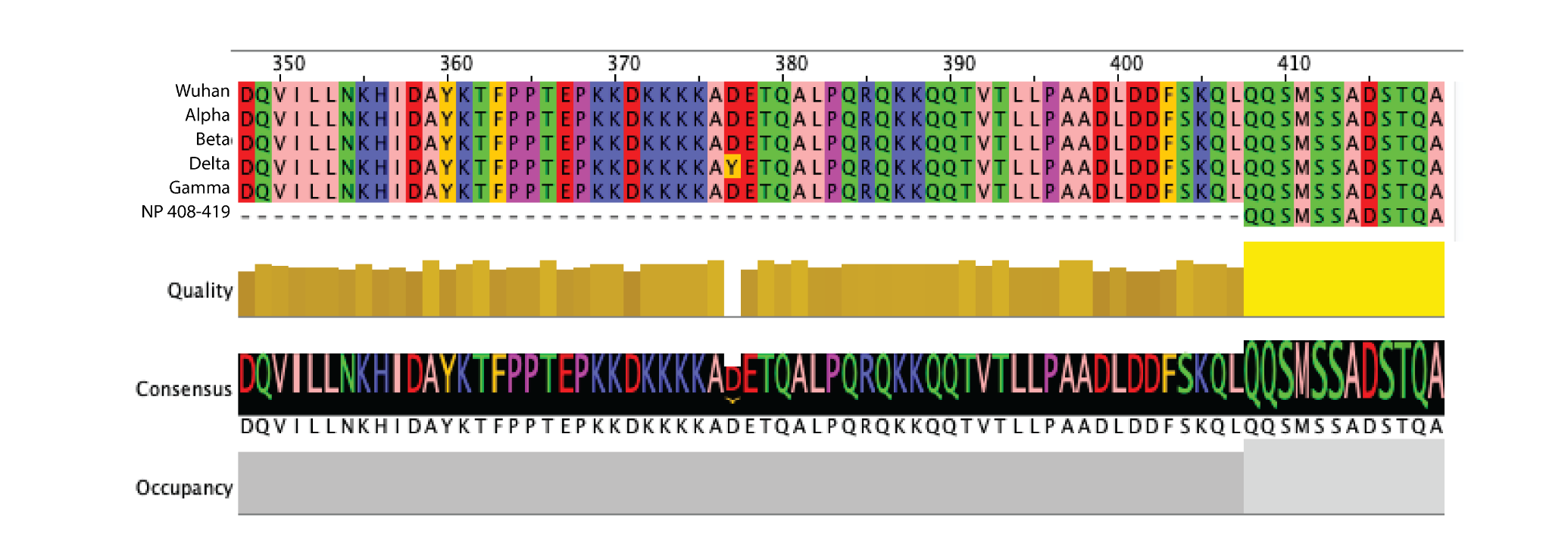
**

**Supplementary Figure 2.** Comparison of predicted nucleoprotein epitopes, from original Wuhan strain to SARS-CoV-2 variants of concern: Alpha, Beta, Gamma and Delta. Quality refers to the alignment quality based on blosum2 algorithm scores, Consensus indicates the abundance of the amino acids present in a particular position and Occupancy is the number of aligned positions. (A) NP 1-51, (B) NP58-85, (C) NP115-127, (D) NP232-269, (E) NP273-287, (F) NP338-347, (G) NP361-390, (H) NP 408-419

**A**
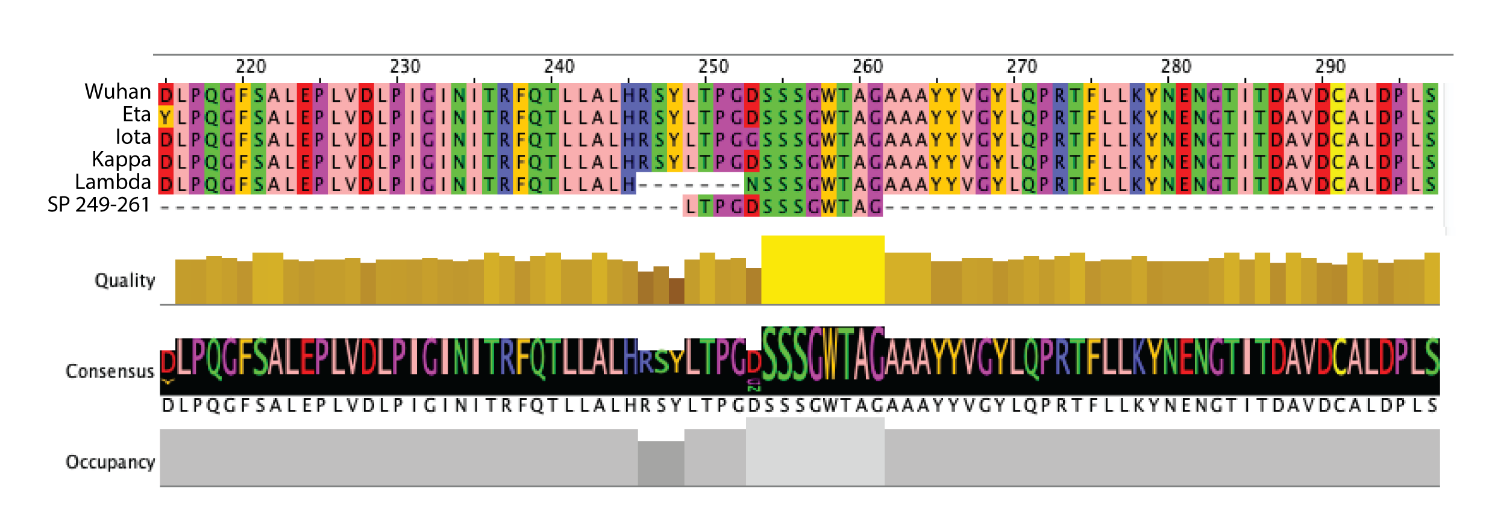


**B**
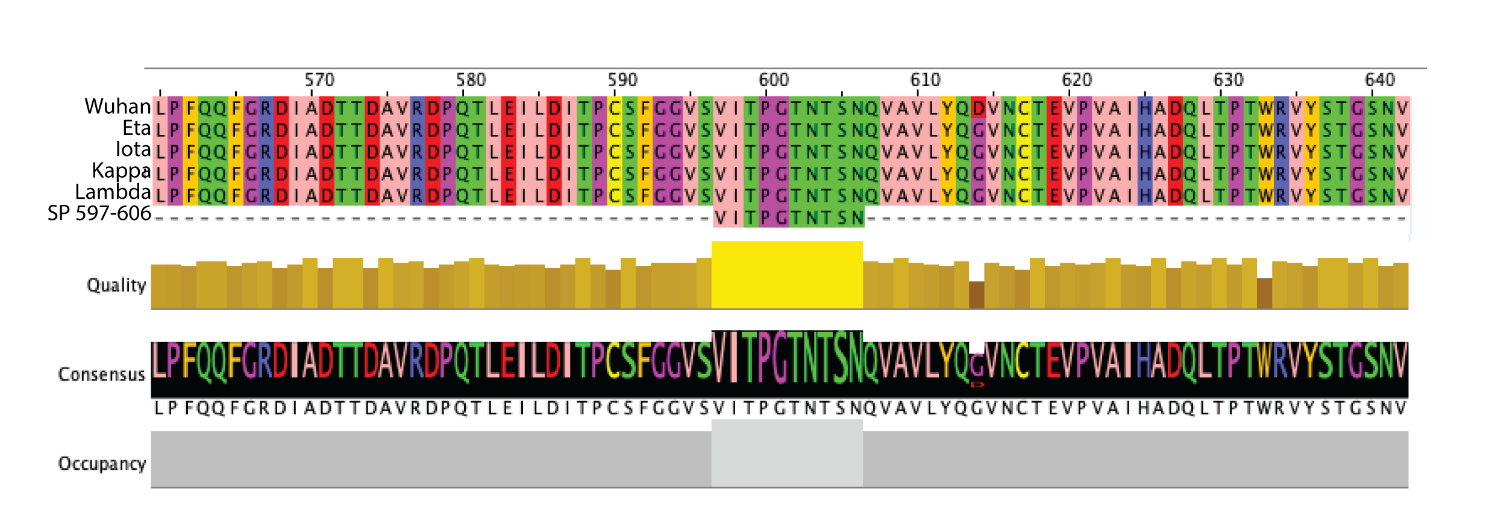


**C**
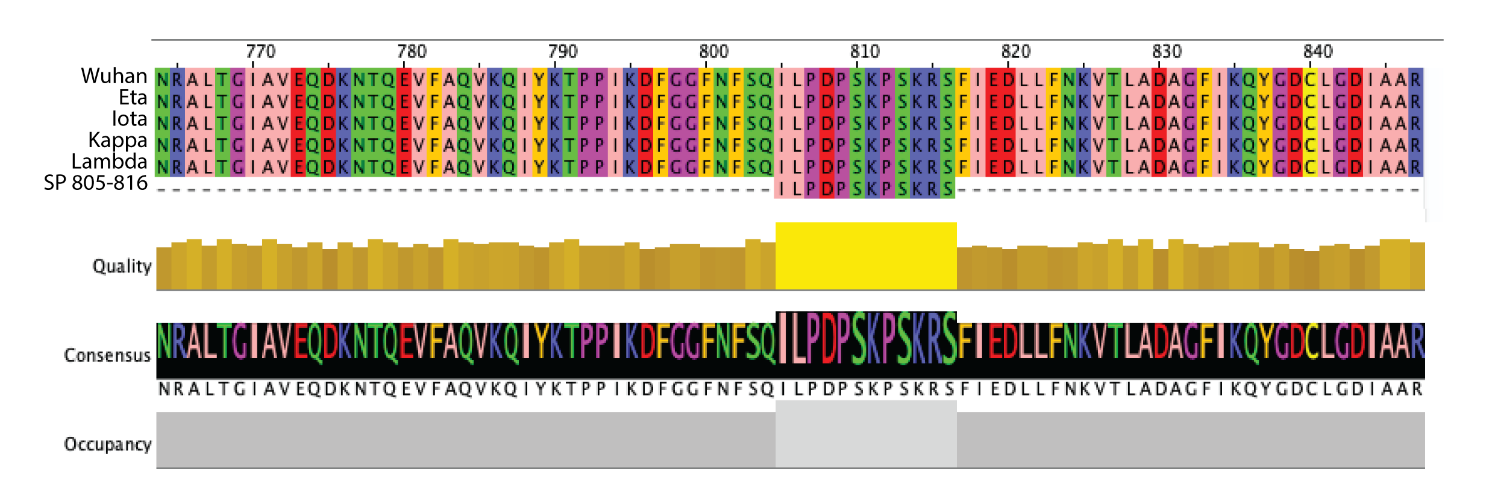


**D**


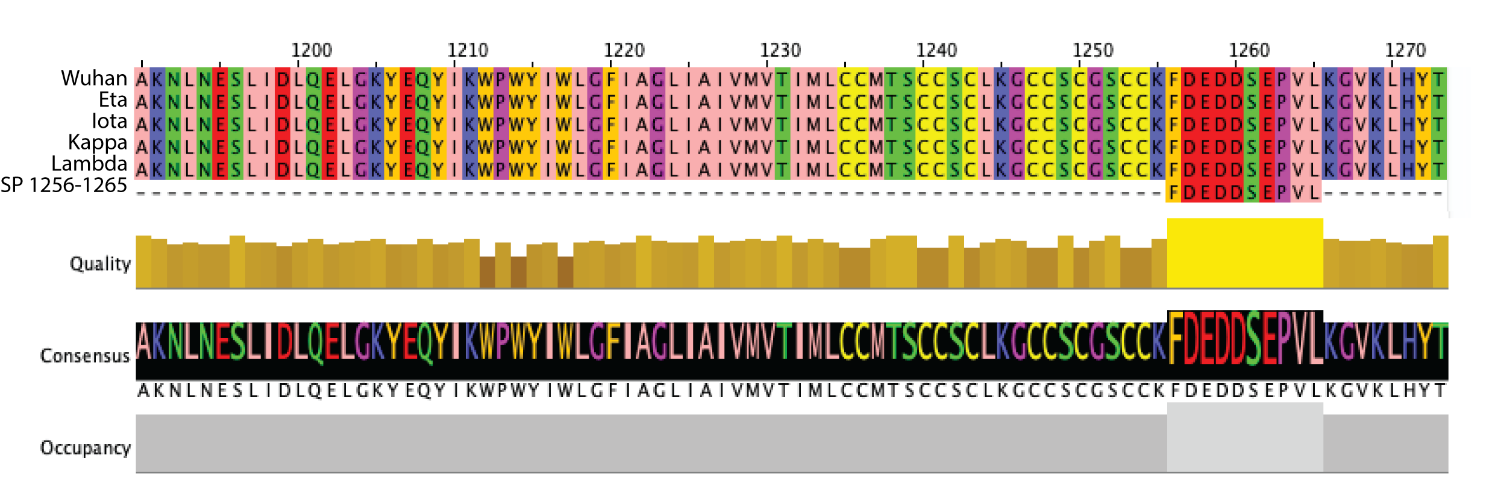


**Supplementary Figure 3.** Comparison of predicted spike epitopes, from original Wuhan strain to SARS-CoV-2 variants of concern: Eta, Iota, Kappa and Lambda. Quality refers to the alignment quality based on blosum2 algorithm scores, Consensus indicates the abundance of the amino acids present in a particular position and Occupancy is the number of aligned positions. (A) SP249-261 (B) SP597-606 (C) SP805-816 (D) SP1256-1265.

**A**


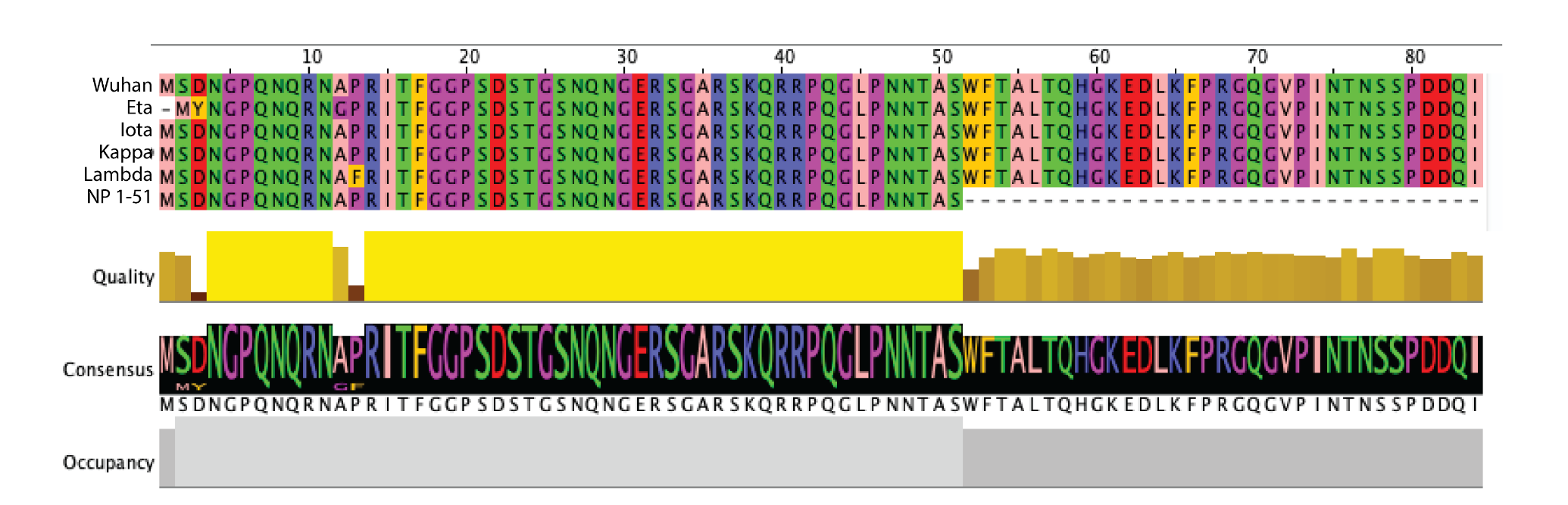


**B**
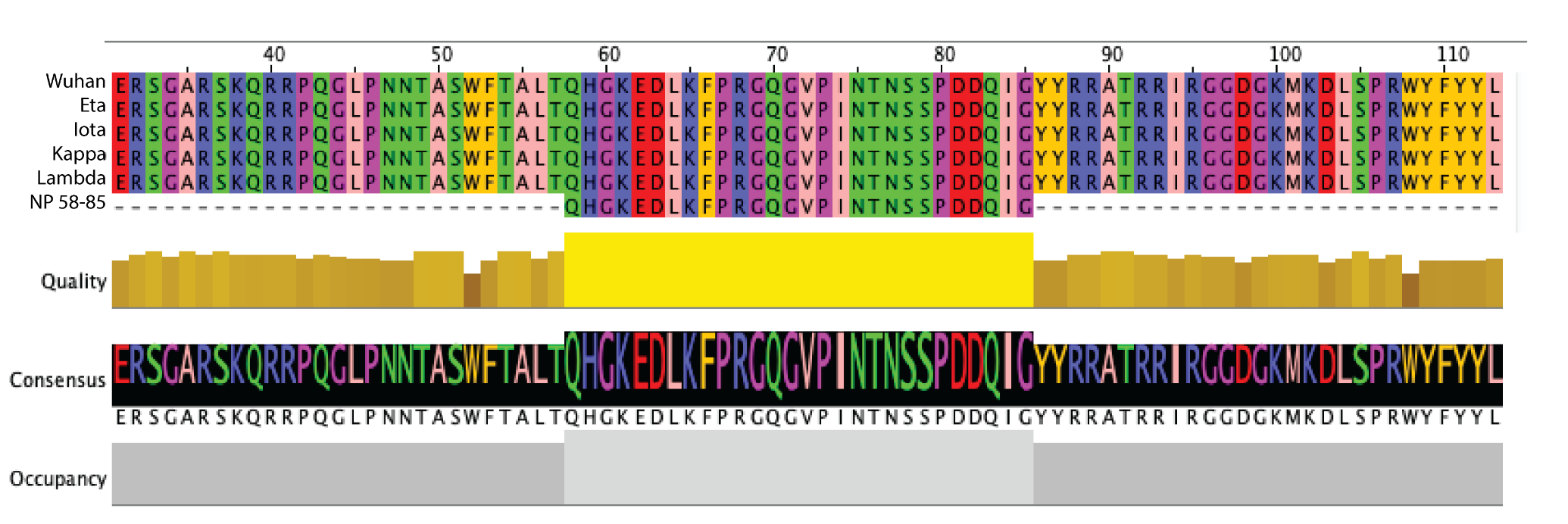


**C**
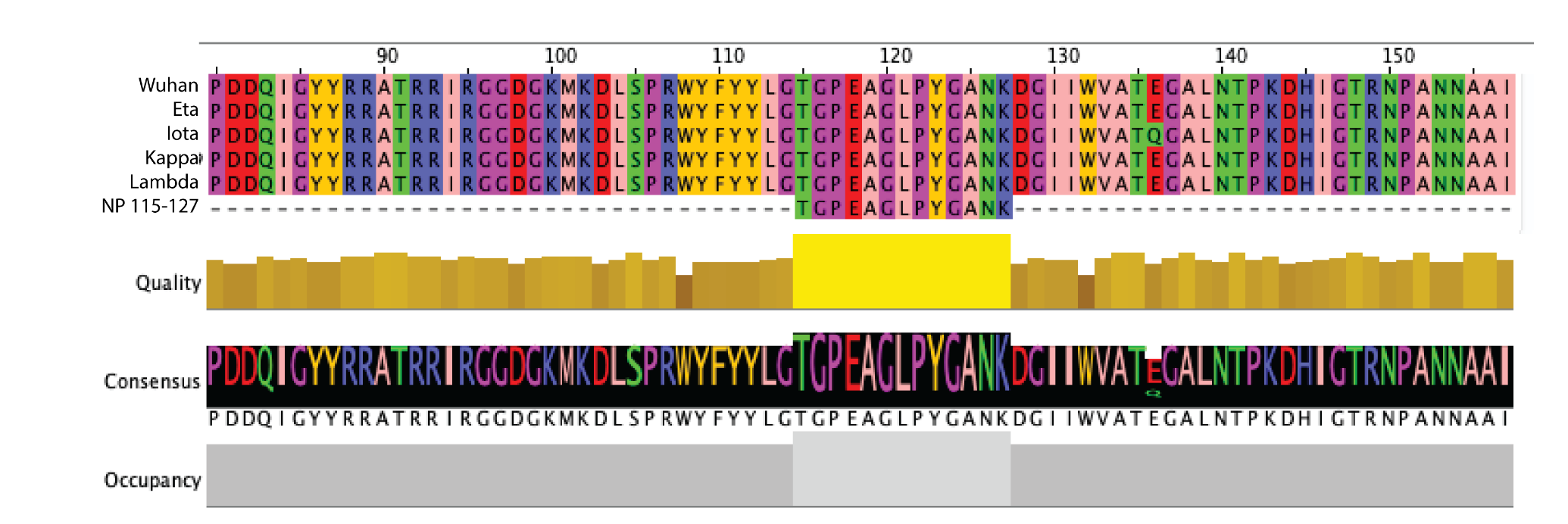


**D**
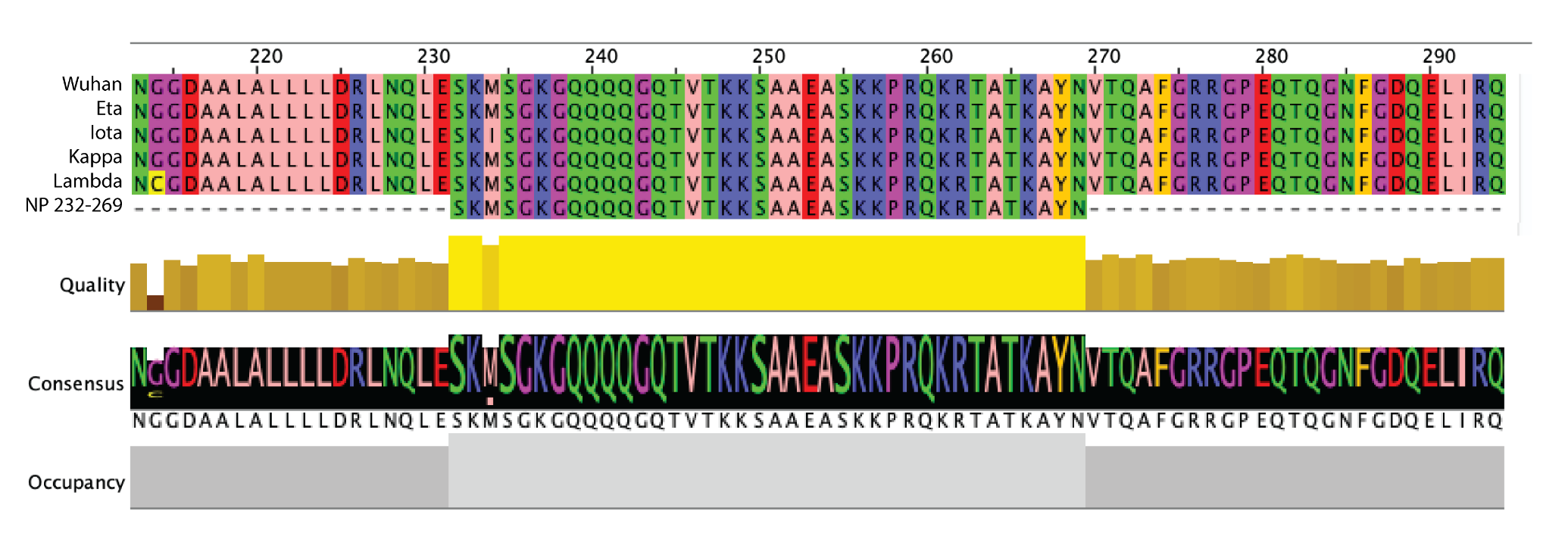


**E**
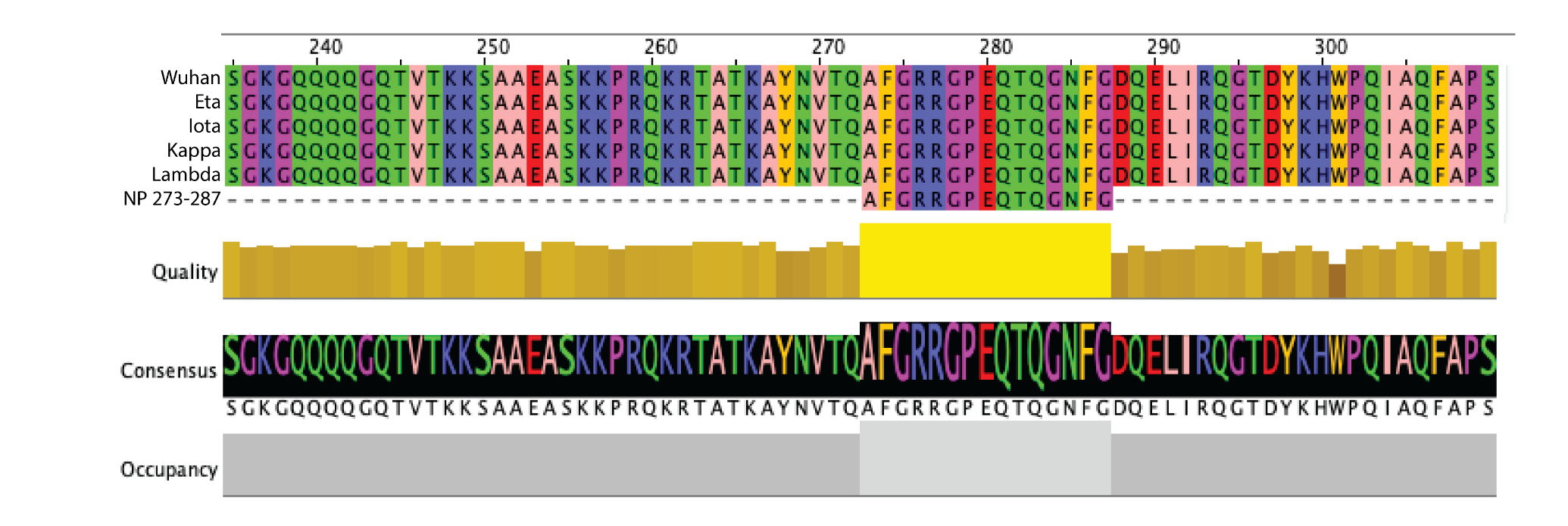


**E**
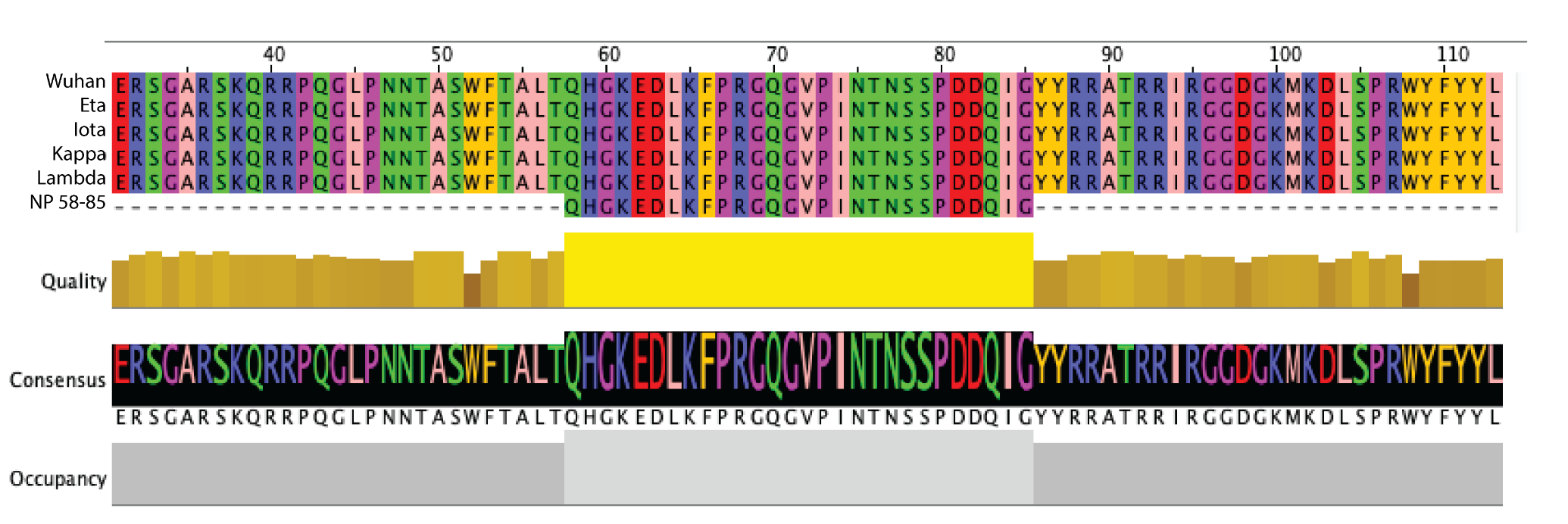


**F**
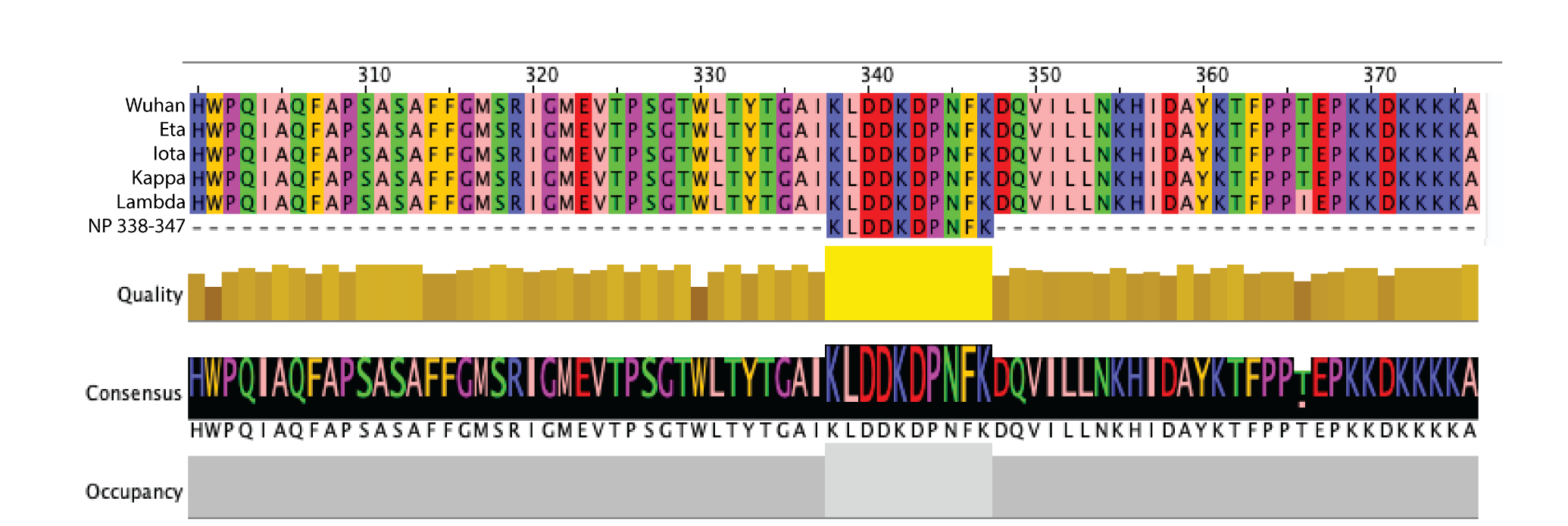


**G**
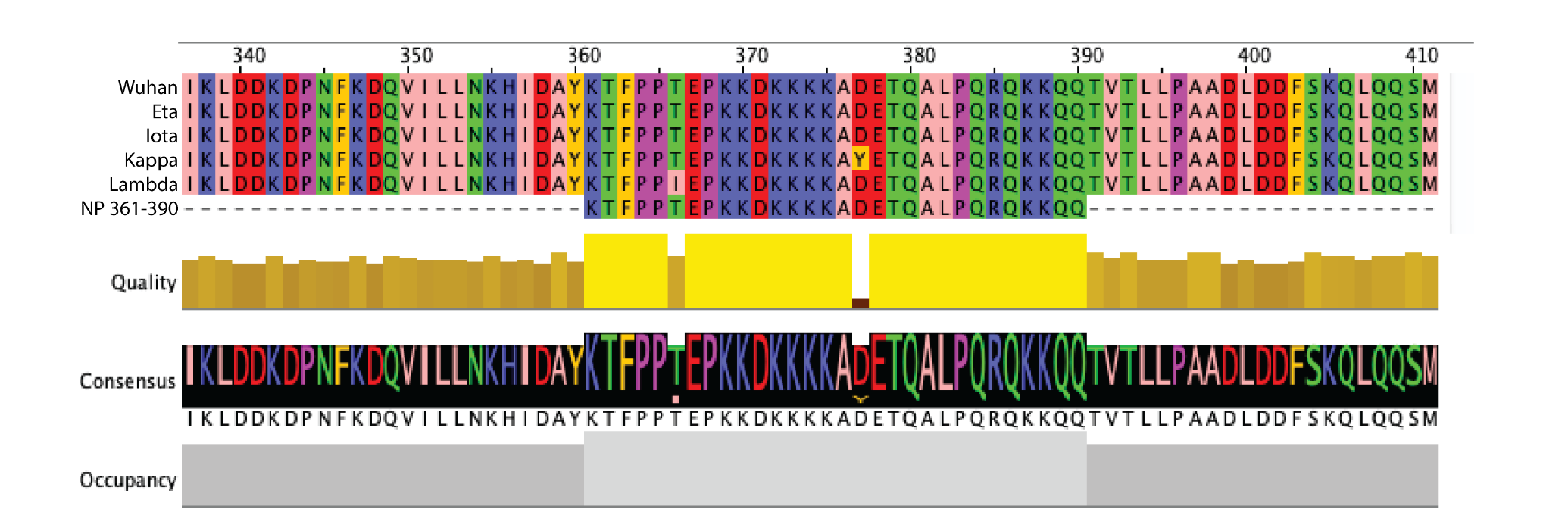


**H**
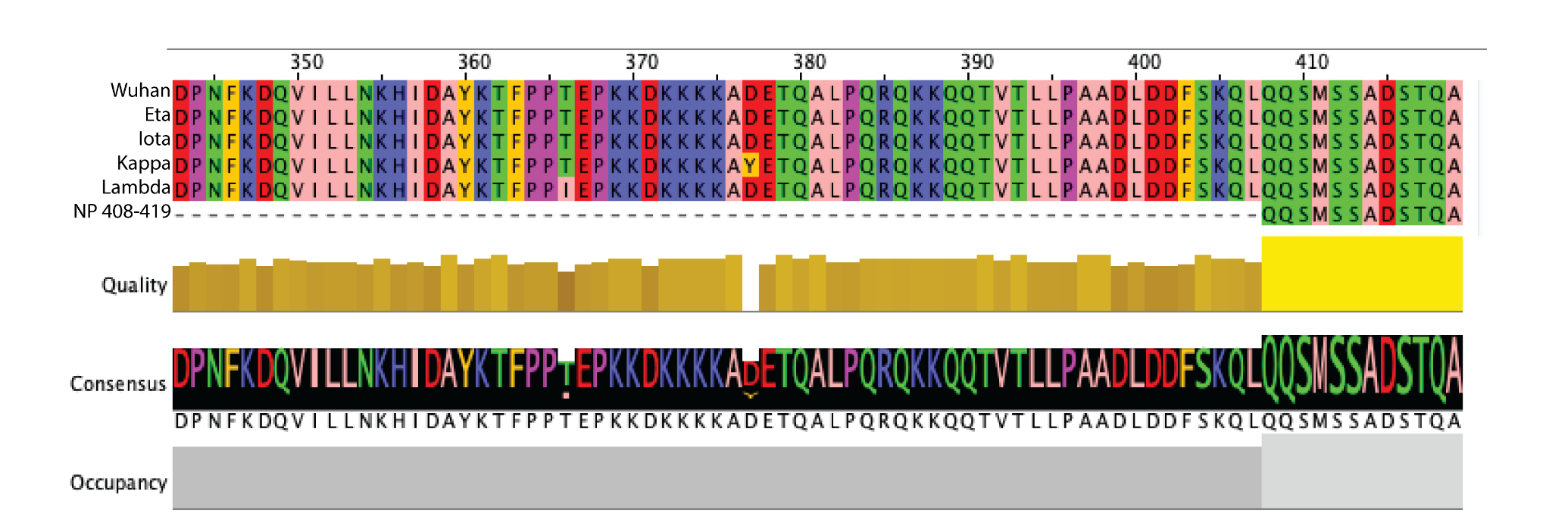


**Supplementary Figure 4.** Comparison of predicted nucleoprotein epitopes, from original Wuhan strain to SARS-CoV-2 variants of interest: Eta, Iota, Kappa and Lambda. Quality refers to the alignment quality based on blosum2 algorithm scores, Consensus indicates the abundance of the amino acids present in a particular position and Occupancy is the number of aligned positions. (A) NP 1-51, (B) NP58-85, (C) NP115-127, (D) NP232-269, (E) NP273-287, (F) NP338-347, (G) NP361-390, (H) NP 408-419

.
